# Exome sequencing reveals variants in known and novel candidate genes for severe sperm motility disorders

**DOI:** 10.1093/humrep/deab099

**Published:** 2021-06-05

**Authors:** M S Oud, B J Houston, L Volozonoka, F K Mastrorosa, G S Holt, B K S Alobaidi, P F deVries, G Astuti, L Ramos, R I Mclachlan, M K O’Bryan, J A Veltman, H E Chemes, H Sheth

**Affiliations:** 1 Department of Human Genetics, Donders Institute for Brain, Cognition and Behavior, Radboud University Medical Center, Nijmegen, The Netherlands; 2 School of Biological Sciences, Monash University, Monash, Australia; 3 School of BioSciences, Faculty of Science, The University of Melbourne, Parkville, Australia; 4 Scientific Laboratory of Molecular Genetics, Riga Stradins University, Riga, Latvia; 5 Biosciences Institute, Faculty of Medical Sciences, Newcastle University, Newcastle upon Tyne, UK; 6 Department of Gynaecology and Obstetrics, Radboud University Medical Center, Nijmegen, The Netherlands; 7 Hudson Institute of Medical Research, Monash University, Clayton, Melbourne, Australia; 8 Centro de Investigaciones Endocrinológicas “Dr. César Bergadá” CEDIE-CONICET-FEI, Hospital de Niños Ricardo Gutiérrez, Buenos Aires, Argentina; 9 Foundation for Research in Genetics and Endocrinology, Institute of Human Genetics, Ahmedabad, India

**Keywords:** whole exome sequencing, dysplasia of the fibrous sheath, multiple morphological abnormalities of the sperm flagella, asthenozoospermia, male infertility, sperm motility disorders, candidate novel genes

## Abstract

**STUDY QUESTION:**

What are the causative genetic variants in patients with male infertility due to severe sperm motility disorders?

**SUMMARY ANSWER:**

We identified high confidence disease-causing variants in multiple genes previously associated with severe sperm motility disorders in 10 out of 21 patients (48%) and variants in novel candidate genes in seven additional patients (33%).

**WHAT IS KNOWN ALREADY:**

Severe sperm motility disorders are a form of male infertility characterised by immotile sperm often in combination with a spectrum of structural abnormalities of the sperm flagellum that do not affect viability. Currently, depending on the clinical sub-categorisation, up to 50% of causality in patients with severe sperm motility disorders can be explained by pathogenic variants in at least 22 genes.

**STUDY DESIGN, SIZE, DURATION:**

We performed exome sequencing in 21 patients with severe sperm motility disorders from two different clinics.

**PARTICIPANTS/MATERIALS, SETTING, METHOD:**

Two groups of infertile men, one from Argentina (n = 9) and one from Australia (n = 12), with clinically defined severe sperm motility disorders (motility <5%) and normal morphology values of 0–4%, were included. All patients in the Argentine cohort were diagnosed with DFS-MMAF, based on light and transmission electron microscopy. Sperm ultrastructural information was not available for the Australian cohort. Exome sequencing was performed in all 21 patients and variants with an allele frequency of <1% in the gnomAD population were prioritised and interpreted.

**MAIN RESULTS AND ROLE OF CHANCE:**

In 10 of 21 patients (48%), we identified pathogenic variants in known sperm assembly genes: *CFAP43* (3 patients); *CFAP44* (2 patients), *CFAP58* (1 patient), *QRICH2* (2 patients), *DNAH1* (1 patient) and *DNAH6* (1 patient). The diagnostic rate did not differ markedly between the Argentinian and the Australian cohort (55% and 42%, respectively). Furthermore, we identified patients with variants in the novel human candidate sperm motility genes: *DNAH12*, *DRC1*, *MDC1*, *PACRG*, *SSPL2C* and *TPTE2*. One patient presented with variants in four candidate genes and it remains unclear which variants were responsible for the severe sperm motility defect in this patient.

**LARGE SCALE DATA:**

N/A

**LIMITATIONS, REASONS FOR CAUTION:**

In this study, we described patients with either a homozygous or two heterozygous candidate pathogenic variants in genes linked to sperm motility disorders. Due to unavailability of parental DNA, we have not assessed the frequency of *de novo* or maternally inherited dominant variants and could not determine the parental origin of the mutations to establish in all cases that the mutations are present on both alleles.

**WIDER IMPLICATIONS OF THE FINDINGS:**

Our results confirm the likely causal role of variants in six known genes for sperm motility and we demonstrate that exome sequencing is an effective method to diagnose patients with severe sperm motility disorders (10/21 diagnosed; 48%). Furthermore, our analysis revealed six novel candidate genes for severe sperm motility disorders. Genome-wide sequencing of additional patient cohorts and re-analysis of exome data of currently unsolved cases may reveal additional variants in these novel candidate genes.

**STUDY FUNDING/COMPETING INTEREST(S):**

This project was supported in part by funding from the Australian National Health and Medical Research Council (APP1120356) to M.K.O.B., J.A.V. and R.I.M.L., The Netherlands Organisation for Scientific Research (918-15-667) to J.A.V., the Royal Society and Wolfson Foundation (WM160091) to J.A.V., as well as an Investigator Award in Science from the Wellcome Trust (209451) to J.A.V. and Grants from the National Research Council of Argentina (PIP 0900 and 4584) and ANPCyT (PICT 9591) to H.E.C. and a UUKi Rutherford Fund Fellowship awarded to B.J.H.

## Introduction

**Figure 1.**
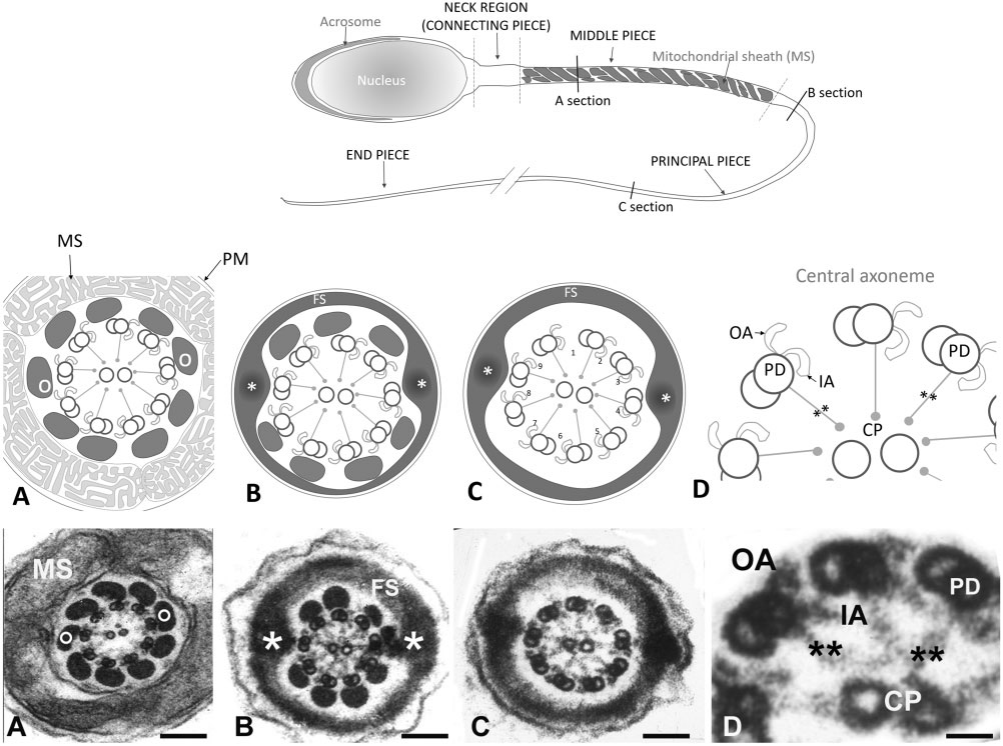
**Structure of the tail of a normal human spermatozoon**. Upper panel: schematic drawing of a normal human spermatozoon showing three consecutive sections along the length of the tail: middle piece, principal piece and end piece. Transversal lines along its length mark the level of the cross sections displayed in the schematic panel drawings (middle panel) and the electron microscope images (lower panel): (**A**) mid piece, (**B**) proximal principal piece, (**C**) distal principal piece and (**D**) higher magnification detail of the axoneme. Panel A: schematic drawings and sections of the mid piece: circumferential to the axoneme there are nine outer dense fibres (o) each associated to the corresponding peripheral pair. They are surrounded by a helically arranged mitochondrial sheath (MS). At the proximal principal piece (Panel B) mitochondria are replaced by the Fibrous Sheath (FS), which is organised in two longitudinal columns (*) that replace outer dense fibres 3 and 8 and are joined by transverse hemi-circumferential ‘ribs’ (FS). At the distal principal piece (Panel C) all outer dense fibres disappear and the axoneme is only surrounded by the Fibrous Sheath. Panels D: higher magnification details of three peripheral doublet microtubules (PD) projecting in a clockwise direction toward the next PD OA and IA dynein arm, and radial spokes (**) towards the central pair. Magnification bars: (A) 150 nm, (B) 140 nm, (C) 104 nm, and (D) 26 nm.

The presence of motile sperm is an absolute requirement for male fertility in all mammals. The sperm flagellum is a modified motile cilium and structural and functional deficiencies of this structure are frequently associated with male infertility (Toure *et al*., 2020). A normal human sperm tail is composed of a central axoneme consisting of nine peripherally arranged doublet microtubules encircling a central pair (Fig. 1). Each peripheral doublet projects towards the next doublet in a clockwise direction, via the presence of outer and inner dynein arms (ODAs and IDAs), which are the key effector structures underpinning sperm motility. The absence of ODAs and/or IDAs of respiratory cilia and sperm flagella in men leads to sperm immotility and chronic respiratory disease in a syndrome collectively known as primary ciliary dyskinesia (PCD, Afzelius *et al*., 1975; Rebbe and Pedersen, 1975; Rossman *et al*., 1981). Key to axoneme function and PCD causality are the dynein complexes within the IDA and ODA, which are ATPases responsible for microtubule sliding within the axoneme of the sperm tail and respiratory cilia. Consistent with the assembly of the sperm tail in a distinct cytoplasmic lobe devoid of protein translation, the loss of function of genes associated with protein transport can lead to sperm motility defects in animal models, spanning all aspects of sperm ultrastructure (Pleuger *et al*., 2020).

Within the broad spectrum of sperm immotility disorders, a range of pathological sub-types exist. In 1987, Chemes *et al.* introduced the term dysplasia of the fibrous sheath (DFS) to describe a distinct form of human sperm pathology involving axonemal and peri-axonemal structures. This condition can be familial, suggesting a genetic aetiology, and/or can be associated with chronic respiratory disease due to dynein deficiency, suggesting a genetic link as well as a mechanistic overlap with PCD (Afzelius *et al*., 1975; Baccetti *et al*., 1981; Chemes *et al*., 1987; Chemes *et al*., 1990; Chemes *et al*., 1998; Rawe *et al*., 2002). In 2014, Ben Khelifa *et al.* introduced the term multiple morphological abnormalities of the sperm flagellum (MMAF) to describe a similar combination of sperm phenotypes (Ben Khelifa *et al*., 2014). Whilst the phenotypes identified as DFS and MMAF are overlapping, the main differences reside in the significance put on short and thick tails (‘stumpy tails’) due to the fibrous sheath disorganisation and associated axonemal anomalies (DFS), and on the relevance given to a lack of the central pair of microtubules or dynein arms (MMAF). In the present paper, we will use the term spectrum of severe sperm motility disorders to include both DFS and MMAF but also the more general description of patients with non-syndromic severe astheno-teratozoospermia.

Unbiased next-generation sequencing methods such as exome sequencing have proven critical in the discovery of the genetic causes underlying severe sperm motility disorders, including variants in Dynein Axonemal Heavy Chain 1 and 6 (*DNAH1* and *DNAH6)* (Ben Khelifa *et al*., 2014; Tu *et al*., 2019), Cilia And Flagella Associated Protein 43 and 44 (*CFAP43* and *CFAP44)* (Tang *et al*., 2017) and Glutamine Rich 2 (*QRICH2*) (Shen *et al*., 2019). Studies in the past 6 years have associated variants in at least 22 genes with severe sperm motility defects and demonstrated that, indeed, a large portion of these defects are genetic in origin (Supplementary Table SI). Currently 30–60% of all DFS-MMAF cases can be explained genetically (Toure *et al*., 2020). In the present study, we aimed to determine the diagnostic value of the currently known sperm motility genes in different clinical cohorts of patients with severe sperm motility defects using an unbiased exome sequencing approach. This also allowed us to identify novel candidate genes involved in sperm morphology and motility.

## Materials and methods

### Patients and sample collection

The current study included two groups of patients, one from Argentina and a second from Australia. All patients were informed of the nature of the study and gave informed consent before collection of blood samples. The collection of samples in Argentina was approved by the Ethics Review Board of Centro de Investigaciones Endocrinológicas, National Research Council, Buenos Aires, Argentina. The collection of samples in Australia was approved by the human ethics panels at three sites: Monash Surgical Private Hospital (Clayton), Monash Medical Centre and Monash University, Australia.

Nine males from Argentina were included, who presented with primary infertility due to severe sperm tail defects and very low motility or immotile sperm (Table I). In addition to the standard semen analysis, their sperm were examined by electron microscopy. All patients were characterised as having a typical DFS-MMAF phenotype (Chemes *et al*., 1987, 1998). ARG5 has a non-twin brother with DFS, while ARG7 and ARG8 have suffered from chronic respiratory disease and sinusitis since early childhood. ARG6 had a combination of DFS-MMAF with ‘acephalic spermatozoa’, a phenotype derived from a faulty development of the sperm head-tail attachment (Rawe *et al*., 2002, Moretti *et al*., 2011).

**Table I:** Light and electron microscopy characteristics of spermatozoa in patients of the Argentinean cohort.

Sample	Shape of tails	Motility: total/ translative	Fibrous sheath thickening	Axoneme**	Dynein arms	Mid piece anomaly	Extension ODFs 3 and 8	Observations
ARG1	Stump*	2/1	Present	8 + 0	Present	Present	Present	Oligozoospermia
ARG2	Stump	0	Present	9 + 0	NE	Present	Present	Oligozoospermia
ARG3	Stump	0	Present	9 + 0 9 + 2	NE	Present	Present	–
ARG4	Stump	NE	Present	NE	NE	NE	NE	Astheno-teratozoospermia.
ARG5	Stump	0	Present	9 + 0	Partial absence	NE	NE	Brother with DFS
ARG6	Stump	0	Present	9 + 0	Present	Present	Present	Combined with acephalic sperm
ARG7	Stump	0	Present	9 + 2	Present	Absent	Absent	Chronic respiratory disease
ARG8	Stump	0	Present	9 + 1 + 1***	Absent	Partial	Partial (?)	Chronic respiratory disease
ARG9	Stump	0	Present	TAD	Absent	NE	NE	Oligozoospermia

* Stump tails: short, thick, of irregular outline.

** Axoneme: 1st digit: number of peripheral doublets, 2nd digit: number of central microtubules.

*** 9 peripheral doublets + 1 centrally translocated peripheral doublet + 1 central microtubule.

NE, not evaluated because of technical limitations; TAD, Total Axonemal Disruption.

Mid piece anomaly: Short or absent mid pieces due to lack of annulus migration.

Extension ODF 3 and 8: ODF 3 and 8 abnormally extended beyond the mid piece.

In the Australian group, 12 males were recruited following assessment of their semen samples via WHO criteria (World Health Organization, 2010). All presented with infertility due to severe asthenozoospermia and a high percentage of abnormal forms, based on light microscopy as reported by the clinical andrology laboratory (Table II). Specifically, these men had sperm motility values <5% and normal morphology values of 0–4%. Patient AUS3 had a history of chronic sinus congestion with productive cough, suggestive of a ciliary defect. Patient AUS3 also experienced respiratory distress of presumed, but unexplored, environmental origin.

**Table II:** Spermiogram and clinical outcome of Australian cohort.

Sample	**Concentration** (x10^6^/ml)*	**Motility** (% motile)*	**Morphology** (% abnormal)*	Additional relevant clinical notes	Fertility outcome after ART treatment
**AUS1**	120	4	95		NA
**AUS2**	20	1	94		NA
**AUS3**	5	5	94	Chronic sinus congestion with productive cough	3 ICSI cycles 2 transferred 0 pregnancies
**AUS4**	29	0	91-95		NA
**AUS5**	15	2	98		NA
**AUS6**	12	0	98		NA
**AUS7**	12	3	96		NA
**AUS8**	0.5	1	97		2 ICSI cycles 2 pregnancies
**AUS9**	45	0	100	Presumed smoker’s cough. Hematuria	NA
**AUS10**	0.1	0	98		
**AUS11**	8.8	5	99		2 ICSI cycles 1 pregnancy
**AUS12**	1	4	100		10 ICSI cycles 1 foetus loss at 20 weeks 2 pregnancies

* Reference values for normozoospermia according to the World Health Organization: ≥15 × 10^6^ sperm per ml; ≥40% motility (progressive motility and non-progressive motility); ≥4% normal forms.

All 21 patients provided a venous blood sample from which DNA was extracted and kept at −80°C until analysis.

### Transmission electron microscopy

As indicated above, in addition to the standard semen analyses, an aliquot of fresh semen from each of the Argentinian patients was processed for transmission electron microscopy (TEM) according to the methods previously described (Chemes *et al*., 1987, 1998). Briefly, within 30–60 min after ejaculation, when liquefaction was complete, samples were diluted in phosphate buffer. After centrifugation pellets were fixed in situ with EM grade glutaraldehyde in phosphate buffer, followed by post-fixation with osmium tetroxide. Sperm pellets were dehydrated followed by infiltration in propylene oxide-epon-araldite mixture, embedded and subsequently polymerised in pure Epon-Araldite (Pelco International, Fresno, CA, USA). Thin sections exhibiting silver to pale golden interference colours were obtained using a Pelco diamond knife in a RMC-7000 ultramicrotome. These sections were mounted on 300 mesh copper grids, double-stained with uranyl acetate and lead citrate, and studied and photographed in a Zeiss 109 electron microscope (Zeiss Oberkochen, Jena, Germany).

### Whole exome sequencing

Samples of 100 ng high-quality genomic DNA, measured with Qubit dsDNA HS kit (Invitrogen, Carlsbad, CA, USA), were used for whole exome target capture using Illumina’s TruSeq Rapid Exome Capture kit (Illumina, San Diego, CA, USA), according to the manufacturer’s protocol. Sample libraries were dual indexed using Illumina’s Nextera i7 and i5 primers (Illumina, San Diego, CA, USA). Pooled libraries were sequenced on the NextSeq 500 platform for the Argentinian cohort (Illumina, San Diego, CA, USA) and the NovaSeq 6000 platform for the Australian cohort (Illumina, San Diego, CA, USA). Paired-end sequencing of 150 bp was carried out at an average sequencing depth of 100× per sample. Whole exome sequencing was carried out at the Genomics Core Facility, Biosciences Institute, Faculty of Medical Sciences, Newcastle University, UK.

FASTQ files were aligned against the human reference genome (hg19/GCRh37) using Burrows Wheeler Aligner (BWA MEM 0.7.12) to generate BAM files. Picard toolkit v1.90 was used to mark PCR duplicates and SAMtools v1.6 was used to sort and index BAM files. Genome Analysis Toolkit (GATK) v3.4.46 was used to perform base quality score recalibration and variant calling to generate gVCF file containing SNVs and small indels for each sample. All gVCF files were annotated using Ensembl’s Variant Effect Predictor (VEP v92) tool. Homozygosity calling was performed using RareVariantVis (Stokowy *et al*., 2016) and regions of > 1 000  000 bp and a percentage of homozygosity larger than 85 (perc_HMZ >85) were classified as stretches of homozygosity.

### Variant filtering, prioritisation and validation

For variant filtering and prioritisation, we focused on variants present in exons and canonical splice sites. Variants were excluded from downstream analysis if they did not meet all of the following criteria: (a) variant was more than five reads covering the locus; (b) variant was present in more than 15% of reads covering that locus; and (c) variant had an allele frequency of <1% in the gnomAD database (https://gnomad.broadinstitute.org), dbSNP (https://www.ncbi.nlm.nih.gov/SNP/) and our internal database. Variants were classed as homozygous if the variant allele was detected in >85% of all reads covering the locus and heterozygous if the variant allele was detected in >15% and <85% of all the reads covering the locus. Following filtering, variants were prioritised based on the following criteria: (a) variants present in known or candidate severe sperm motility disorder genes (*AK7, AKAP4, ARMC2, CEP135, CFAP43, CFAP44, CFAP58, CFAP65, CFAP69, CFAP70, DNAH1, DNAH2, DNAH6, DNAH17, DZIP1, FSIP2, MAATS1, QRICH2, SPEF2, TTC21A, TTC29* and *WDR66*); (b) genes which were mutated in multiple patients; (c) homozygous variants which were present in homozygosity stretches of >1 Mb in length; (d) genes which were reported as having elevated mRNA expression in testis, which is available from the Human Protein Atlas database version 19.1 (https://www.proteinatlas.org/humanproteome/tissue/testis); (e) genes which interact with known sperm motility or cilia related genes in the STRING database version 11.0 (https://string-db.org); and (f) genes which present infertility or astheno-teratozoospermia phenotypes as reported in the Mouse Genomics Institute database (http://www.informatics.jax.org); database last accessed on 8 November 2019 or elsewhere in the literature.

Candidate variants were classified according to the guidelines of the American College of Medical Genetics using five classes: benign (Class 1), likely benign (Class 2), variant of unknown significance (Class 3), likely pathogenic (Class 4) and pathogenic (Class 5) (Richards *et al*., 2015) using the software program Alamut Visual version v.2.13. Missense pathogenicity prediction was performed by Align GVGD, SIFT, MutationTaster and PolyPhen-2 and splicing prediction was performed as described previously (Houston *et al*., 2020). Variants on chromosome X were classified as (likely) benign if the allele frequency in men exceeded 0.05% in any population described in gnomAD. Candidate variants following filtering and prioritisation were visually inspected in the IGV browser (http://software.broadinstitute.org/software/igv/) to evaluate variant quality. Lastly, candidate variants were validated using the conventional Sanger sequencing approach according to the standard protocols.

### Control cohort of proven fathers

To assess the frequency of all variants prioritised in our analysis, we used a control cohort of 5784 proven fathers as described previously (Wyrwoll *et al*., 2019). Detailed information regarding child conception was unavailable for these men, but they likely reflect the normal population of fathers in the Netherlands. Currently, approximately 1 in 33 children in the Netherlands is conceived through any form of IVF, ICSI or transfer of previously frozen embryos, and 1 in 98 is conceived through ICSI alone as reported by the Dutch Society for Obstetrics and Gynecology (https://www.degynaecoloog.nl/nuttige-informatie/ivf-resultaten/).

### CNV analysis

CNVs were detected from the exome sequencing data using a custom GATK4-based pipeline. This workflow exploits the GATK4 sequence read-depth normalisation (McKenna *et al*., 2010) and a custom R-based segmentation and visualisation. The CNVs identified were annotated with AnnotSV3 (https://lbgi.fr/AnnotSV/). Due to low quality of the CNV data, samples from ARG5, ARG3 and AUS9 were excluded from the analysis. The common CNVs identified in more than 1% of the samples of the Database of Genomic Variations were excluded. For the rare and large CNVs encompassing ≥ 20 sequencing probes, the Log2Ratio plots were manually inspected and the genes involved were investigated to find any linked to spermatogenesis and testis function. A panel of known primary ciliary dyskinesia genes comprehensive of 32 (described in Takeuchi *et al*., 2020) as well as the known or candidate severe sperm motility disorder genes reported in Supplementary Table SI were used to screen the genes involved in all of the CNVs detected.

## Results

### Sperm phenotype under light and electron microscopy

**Figure 2.**
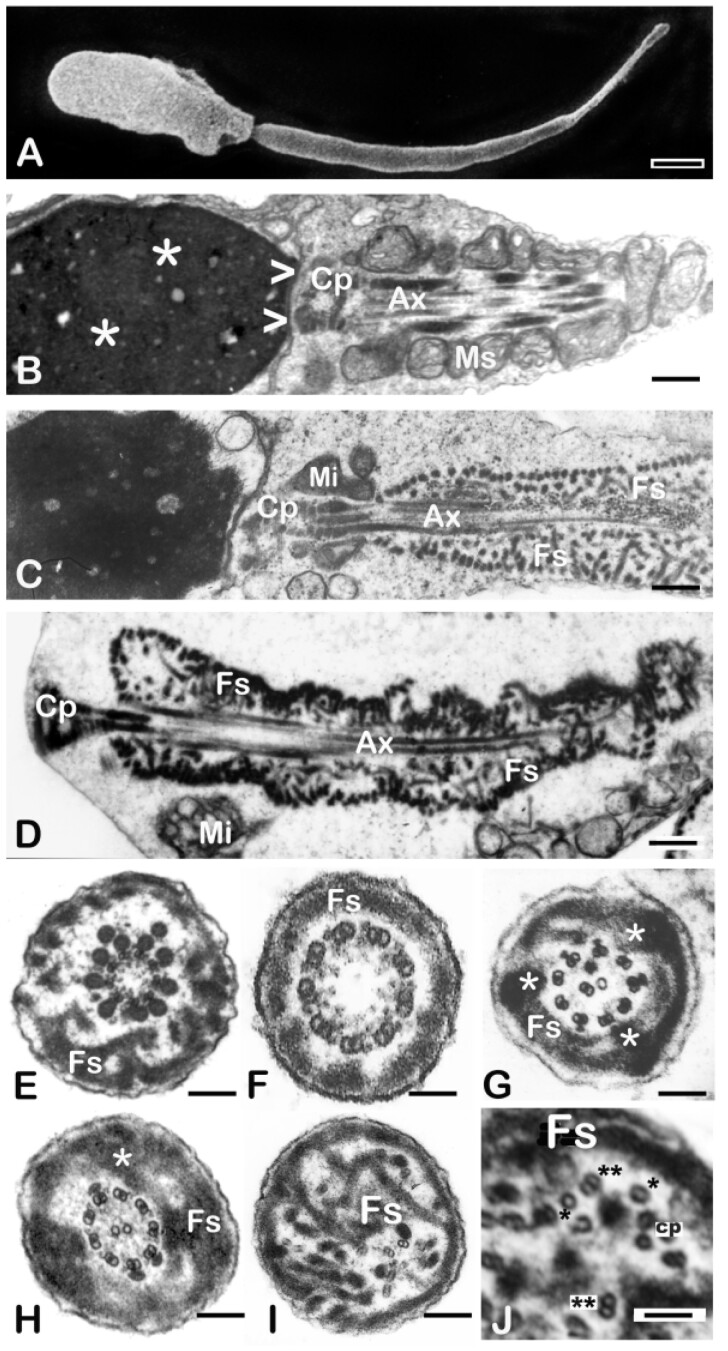
**Characterisation of the DFS-MMAF phenotype by scanning and transmission electron microscopy**. (**A**) Scanning EM of a typical DFS-MMAF spermatozoon (ARG1). The head is abnormally shaped and the mid piece is absent. The sperm tail is very short and thick (7.7 µm long and 630 nm in diameter (normal values of 50 µm in length and 100–140 nm in diameter). (**B**) Longitudinal section of the head, connecting piece and mid piece of a normal human spermatozoon. The sperm head shows densely condensed chromatin (asterisks). At its caudal pole there is a shallow concavity (the implantation fossa, arrowheads) where the connecting piece (CP) and centrioles of the sperm tail are lodged. A helically arranged mitochondrial sheath (MS) surrounds the first part of the axoneme (Ax) with its central microtubules and peripheral outer dense fibers (see mid piece cross section in Fig. 1). (**C**) Longitudinal section of a typical DFS-MMAF spermatozoon to illustrate the details of the phenotype (patient not included in the genetics part of this study). A largely missing mitochondrial sheath is replaced by few mitochondria (Mi). The axoneme (Ax) and outer dense fibers are surrounded by a thick, multilayered, haphazardly arranged fibrous sheath (FS). (**D**) Longitudinal section of a DFS-MMAF/acephalic spermatozoon (ARG6). The connecting piece (CP) takes up the cranial position, there is no mid piece and a misplaced mitochondrion lays besides the flagellum (Mi). A disorganised, multi-layered fibrous sheath (FS) encloses the centrally located axoneme (Ax) and surrounding outer dense fibres. (**E**–**J**) Sperm tail transverse sections of DFS-MMAF spermatozoa showing redundant and disorganised fibrous sheaths (FS). In Panel E (ARG6), the central pair is missing, there are two extra outer dense fibres and the FS is thickened and disorganised. Dynein arms are present. Panel F (ARG5) corresponds to a distal section of the tail principal piece. The axoneme is 9 + 0 (lack of central pair). Dynein arms are present. The FS is not redundant (distal part of the flagellum). In Panel G (ARG8), one microtubule is missing from the central pair and there is central translocation of one supernumerary peripheral doublet. Dynein arms are absent, a superfluous and disorganised FS shows three lateral columns (asterisks). In Panel H (ARG7), the axoneme is normal, and a very thick FS presents with one extra lateral column (asterisk). Panel I (ARG9) shows complete axonemal disruption, a hyperplastic FS and two microtubular pairs lacking dynein arms (down and right from the centre). Panel J (ARG9) shows a higher magnification detail of a DFS tail with disrupted axoneme and hyperplastic FS (Fs). Note a dislocated central pair (cp) and various singlet (*) and doublet microtubules (**) with absent dynein arms. Magnification bars: (A) 1.14 µm, (B) 741 nm, (C) 1012 nm, (D) 533 nm, € 272 nm, (F) 92 nm, (G) 270 nm, (H) 274 nm, (I) 190 nm, and (J) 75 nm.

Sperm from all men in the Argentinian cohort exhibited the DFS-MMAF phenotype, as verified at a light and electron microscopic level (Table I and Fig. 2). The main features of this phenotype include severe astheno-teratozoospermia (<5% motility) or total immotility (Table I). Most spermatozoa present with short, thick and irregular tails (‘stump tails’, Fig. 2A, C and D). There are occasional sperm heads with absent flagella. Ultrastructural examination shows serious architectural disruptions. Thick and short stump tails are packed by disorganised thick filaments corresponding to the ribs of the fibrous sheath and the axonemes depict serious distortions such as partial to complete lack of the central pair (9 + 0 configuration, Fig. 2E and F). Dynein arms (inner or both) are frequently absent from the axoneme peripheral doublets (Fig. 2G, I and J) and, on occasions, the axoneme is completely disrupted (Fig 2I and J). Outer dense fibres 3 and 8 are abnormally extended to the sperm tail principal piece (Table I and Fig. 2E and G). As a consequence of failed caudal migration of the annulus, mitochondria do not assemble properly and the mid piece is missing or substantially reduced to very few mitochondria (Fig. 2A, C and D). Semen samples from the Australian cohort were examined following the WHO 2010 criteria for semen analysis (World Health Organization, 2010) and were characterised as severe astheno-teratozoospermia (Table II).

### Exome sequencing in patients with severe sperm motility disorders

Exome sequencing revealed an average of 92 504 variants per patient (Supplementary Table SII). Since severe sperm motility disorders typically follow an autosomal recessive inheritance pattern, we focussed our analysis on compound heterozygous and homozygous variants, supplemented with an analysis of X and Y-linked variants. After exclusion of false-positive variant calls and variants classified as (likely) benign according to the ACMG guidelines, we identified an average of five variants in each patient for further consideration (Supplementary Tables SII and SIII). Parental data were not available to confirm compound heterozygosity of the heterozygous variants. CNV analysis was performed in all patient exome data, but no clinically relevant CNVs were detected.

In 10 out of 21 patients (47.6%), we found homozygous or 2 heterozygous high confidence disease causing variants in genes previously associated with severe sperm motility disorders (Table III and Supplementary Table SIII): *CFAP43* (3 patients: ARG2, AUS8 and AUS9); *CFAP44* (2 patients: ARG6 and ARG9), *CFAP58* (1 patient: ARG5), *QRICH2* (2 patients: AUS5 and AUS12), *DNAH1* (1 patient: AUS2) and *DNAH6* (1 patient: ARG3). The homozygous variants found in ARG5, AUS8, AUS9 and AUS12 were each located in a region of homozygosity indicating consanguinity (Supplementary Table SIV). None of the variants were found to be present as homozygous in a control cohort of 5784 proven fathers (Supplementary Tables SI and SV).

**Table III:** Selected variants prioritised from the exome sequencing data in severe sperm motility disorders.

Patient	Gene	cDNA*	Protein	Zygosity	GnomAD variant frequency (population with highest frequency)	Variant classification (ACMG)**	Gene expression enriched in testis***	Disease model described	Additional information (see also Supplementary Table SI)	Conclusion
**ARG1**	*DNAH12*	c.5393T>C	p.(Phe1798Ser)	Het	0.1% (AFR: 0.4%)	VUS	Yes	No	Variants have highly similar allele frequencies suggesting they reside on the same allele	Unclear if disease causing
		c.7438C>T	p.(Pro2480Ser)	Het	0.0% (AFR: 0.2%)	VUS				
**ARG2**	*CFAP43*	c.1442 + 1G>A	p.?	Het	0.0% (NFE: 0.0%)	Likely pathogenic	Yes	Yes, mouse (Tang *et al*., 2017)	c.1442 + 1G>A is present in trans with c.1040T>C Known gene c.1040T>C previously reported (Coutton *et al*., 2018)	Probably disease causing
		c.1019T>C	p.(Phe340Ser)	Het (in cis with c.1040T>C)	0.01% (NFE: 0.03%)	Unlikely pathogenic				
		c.1040T>C	p.(Val347Ala)	Het (in cis with c.1019T>C)	0.01% (NFE: 0.02%)	VUS				
**ARG3**	*DNAH6*	c.1316 + 1_1316 + 2insC	N/A	Het	0.0% (NFE: 0.0%)	Likely pathogenic	Yes	Yes, mouse and zebrafish (Li *et al*., 2016)	Known gene	Probably disease causing
		c.7762C>T	p.(Arg2588*)	Het	0.0% (AMR: 0.0%)	Pathogenic				
**ARG4**	*PACRG*	c.369T>A	p.(Tyr123*)	Hom	0.00%	Likely pathogenic	Yes	Yes, mouse (Lorenzetti *et al*., 2004)	Associated with the development of sperm flagellum (Lorenzetti *et al*., 2004; Li *et al*. 2015)	Novel candidate gene
**ARG5**	*CFAP58*	c.1360C>T	p.(Gln454*)	Hom	0.05% (ASJ: 1.09%)	Pathogenic	Yes	No	In homozygosity region. Variant is relatively common in Ashkenazi Jewish population	Probably disease causing
**ARG6**	*CFAP44*	c.652del	p.(Arg218Aspfs*37)	Hom	0.03% (ASJ: 0.61%)	Pathogenic	Yes	Yes, mouse (Tang *et al*., 2017)	Known gene	Disease causing
**ARG7**	*DRC1*	c.238C>T	p.(Arg80*)	Het	0.00% (FIN: 0.03%)	Pathogenic	Yes	Yes, Chlamydomonas reinhardtii (Wirschell *et al*., 2013)	Described in primary ciliary dyskinesia (Wirschell *et al*., 2013; Morimoto *et al*., 2019a)	Novel candidate gene
		c.352C>T	p.(Gln118*)	Het	0.04% (0.07% (NFE)	Pathogenic				
**ARG8**	*DNAH6*	c.2059C>A	p.(Pro687Thr)	Hom	0.04% (NFE: 0.07%)	VUS	Yes	Yes, mouse and zebrafish (Li *et al*., 2016)	Known gene	Possible candidate gene
	*ATP2B4*	c.376G>C	p.(Gly126Arg)	Hom	0.01% (SAS: 0.08%)	VUS	No	Yes, mouse (Schuh *et al*., 2004)	Mouse displays asthenozoospermia. In homozygosity region	Possible candidate gene
	*CEP350*	c.229A>G	p.(Arg77Gly)	Hom	0.34% (ASJ: 0.81%)	VUS	No	No	In homozygosity region	Possible candidate gene
	*CEP290*	c.5998A>G	p.(Ile2000Val)	Het	0.02% (NFE: 0.04%)	VUS	No	Yes, mouse (Lancaster *et al*., 2011)	Described in patients with Leber’s Congenital Amaurosis and asthenozoospermia (Yzer *et al*., 2012)	Possible candidate gene
		c.1092T>G	p.(Ile364Met)	Het	0.08% (SAS: 0.35%)	VUS				
**ARG9**	*CFAP44*	c.2674A>G	p.(Met892Val)	Het	0.05% (AMR: 0.08%)	Likely benign	Yes	Yes, mouse (Tang *et al*., 2017)	Known gene	Probably disease causing
		c.2107A>G	p.(Arg703Gly)	Het	0.00%	VUS				
		c.2104A>T	p.(Ile702Leu)	Het	0.00%	Likely benign				
		c.1174T>C	p.(Trp392Arg)	Het	0.00%	VUS				
**AUS1**	*-*	–	–	–	–	–	–	–	–	No candidate genes found
**AUS2**	*DNAH1*	c.5105G>A	p.(Arg1702Gln)	Het	0.00% (NFE: 0.00%)	VUS	No	Yes, mouse (Neesen *et al*., 2001)	Known gene	Probably disease causing
		c.10823 + 1G>C	p.?	Het	0.00%	Likely pathogenic				
**AUS3**	*-*	–	–	–	–	–	–	–	–	No candidate genes found
**AUS4**	*SPPL2C*	c.634C>T	p.(Arg212Trp)	Hom	0.01% (SAS: 0.06)	VUS	Yes	Yes, mouse (Niemeyer *et al*., 2019)	SPPL2c deficiency leads to a partial loss of elongated spermatids and reduced motility of mature spermatozoa, but preserved fertility in mice (Niemeyer *et al*., 2019). Possibly involved in acrosome formation (Papadopoulou *et al*., 2019)	Novel candidate gene
**AUS5**	*QRICH2*	c.145dup	p.(Thr49Asnfs*31)	Hom	0.00% (NFE: 0.00%)	Pathogenic	Yes	Yes, mouse (Shen *et al*., 2019)	Known gene	Disease causing
**AUS6**	*-*	–	–	–	–	–	–	–	–	No candidate genes found
**AUS7**	*TPTE2*	c.715C>T	p.(Gln239*)	Hom	0.00% (NFE: 0.19%)	Likely pathogenic	Yes	No	Voltage-sensitive phosphatase (Halaszovich *et al*., 2012)	Novel candidate gene
**AUS8**	*CFAP43*	c.335A>T	p.(Asp112Val)	Hom	0.01% (NFE : 0.01%)	VUS	Yes	Yes, mouse (Tang *et al*., 2017)	Known gene In homozygosity region	Probably disease causing
**AUS9**	*CFAP43*	c.944del	p.(Gly315Alafs*22)	Hom	0.00%	Pathogenic	Yes	Yes, mouse (Tang *et al*., 2017)	Known gene. In homozygosity region	Disease causing
**AUS10**	*-*	–	–	–	–	–	–	–	–	No candidate genes found
**AUS11**	*MDC1*	c.472C>T	p.(Gln158*)	Het	0.00%	Likely pathogenic	Yes	Yes, mouse (Lou *et al*., 2006)	Mouse knock-out possibly has a meiotic defect (Lou *et al*., 2006)	Novel candidate gene
		c.2134C>T	p.(Gln712*)	Het	0.00%	Likely pathogenic				
**AUS12**	*QRICH2*	c.169G>A	p.(Glu57Lys)	Hom	0.01% (NFE: 0.02)	VUS	Yes	Yes, mouse (Shen *et al*., 2019)	Known gene. In homozygosity region	Probably disease causing

* gDNA position and transcript information are available in Supplementary Table SIII.

** VUS: Variant of Unknown Significance.

*** Based on the Human Protein Atlas version 19.1.

The full table is available in Supplementary Table SIII.

### Novel candidate genes for severe sperm motility disorders

Expanding the analysis to consider putative variants in genes not previously associated with human astheno-teratozoospermia, revealed a total of 71 variants in 53 genes in the remaining patients (Supplementary Tables SII and SIII). After assessing the predicted pathogenicity of the variant, gene expression pattern in the testis, protein–protein interactions, relevant animal models and previous publications found in PubMed, we classified an additional 11 genes in seven patients as novel or possible candidate gene for a severe motility disorder. All other variants were classified as unlikely to be disease causing (Supplementary Table SIII).

From the Argentinian cohort, ARG1, a patient with typical DFS-MMAF features and no reported symptoms of PCD, carried two heterozygous variants in Dynein Axonemal Heavy Chain 12 (*DNAH12*) (c.5393T>C; p.(Phe1798Ser) and c.7438C>T; p.(Pro2480Ser)) (Table III). *DNAH12* expression is restricted to the ciliated cells in the brain, fallopian tube, lung and testis (Dumur *et al*., 1990). The variant allele frequency of these two variants is very similar in control populations, indicating that they are present on the same allele and may thus not be compound heterozygous. It remains unclear whether variants in *DNAH12* cause DFS-MMAF in this patient. ARG4, carried a homozygous nonsense variant (c.369T>A; p.(Tyr123*)) in Parkin Co-regulated (*PACRG*) (Table III), which has not been described before in public databases such as gnomAD. The variant likely results in nonsense-mediated decay of *PACRG* mRNA. Lastly, in patient ARG7, a man with DFS-MMAF in combination with chronic respiratory disease, we identified two heterozygous nonsense variants (c.238C>T; p.(Arg80*)) in exon 2 and (c.352C>T; p.(Gln118*)) in exon 3 in Dynein Regulatory Complex Subunit 1 (*DRC1*) (Table III). DFS-MMAF patient ARG8 carried variants in multiple candidate genes previously associated with ciliated cell development: *DNAH6*, *ATP2B4*, *CEP350* and *CEP290*.

The Australian patient AUS4 carried a homozygous missense variant (c.634C>T; p.(Arg212Trp)) in Signal Peptide Peptidase Like 2C (*SPPL2C*) (Table III). Patient AUS7 carried a homozygous nonsense variant (c.715C>T; p.(Gln239*)) in Transmembrane Phosphoinositide 3-Phosphatase and Tensin Homolog 2 (*TPTE2*), which has a highly testis enriched expression pattern. Finally, we identified two heterozygous nonsense variants (c.472C>T; p.(Gln158*) in Exon 3 and c.2134C>T; p.(Gln712*) in Exon 7) in Mediator of DNA Damage Checkpoint 1 (*MDC1*) in patient AUS11, who suffered from mild oligozoospermia combined with astheno-teratozoospermia (Table III). In humans, *MDC1* is detected in all tissues but is most strongly expressed in the testis (Uhlen *et al*., 2010; GTEx Consortium, 2015).

### Analysis of homozygous loss-of-function variants in proven fathers

In our analysis of 21 patients, we identified four patients with a homozygous loss-of-function (LoF) variant in a gene known to be required for normal sperm tail assembly and function. Given the large number of genes involved in sperm tail assembly, we assessed whether sequencing a control cohort of 5784 proven fathers would result in a similar number of homozygous LoF variants. In all 22 known sperm tail assembly genes as well as the 6 new candidate genes, one homozygous LoF carrier was identified among the 5784 proven fathers (Supplementary Tables SI and SV). This variant was (NM_001039706.2: c.992del; p.(Gly331Alafs*6)) in Cilia And Flagella Associated Protein 69 (*CFAP69)*.

## Discussion

With the recent application of exome sequencing to previously unexplained individuals with severe sperm tail assembly disorders, variants in at least 22 genes have now been implicated in the spectrum of motility disorders due to tail abnormalities (Supplementary Table SI). In this study, we set out to find the causative genetic variants in known and novel candidate genes in 21 men suffering from severe astheno-teratozoospermia. In 10 out of 21 patients (47.6%), we identified pathogenic or likely pathogenic mutations in a total of 6 known severe sperm motility disorder genes, *CFAP43* (n = 3), *CFAP44* (n = 2), *CFAP58* (n = 1), *QRICH2* (n = 2), *DNAH1* (n = 1) and *DNAH6* (n = 1). In addition, we identified predicted pathogenic mutations in novel candidate genes in seven other patients (33%).

### Exome sequencing is an effective method to identify genetic causes of severe motility disorders

With the use of exome sequencing, we demonstrated that variants in known sperm motility genes likely explained the disorder in 10/21 individuals, reaching a diagnostic yield of approximately 48%. This result is in concordance with previous estimates for the severe sperm motility disorder DFS-MMAF (Coutton *et al*., 2019; Shen *et al*., 2019). Interestingly, three patients in our cohort carried variants in the recently discovered genes *CFAP58* (He *et al*., 2020), *QRICH2* (Shen *et al*., 2019) and *DNAH6* (Tu *et al*., 2019), further supporting their role in causing DFS-MMAF. Exome sequencing is therefore a highly efficient method for genetic diagnostics in patients with defective sperm motility disorders. Of note, while the two cohorts included in this study were collected and phenotyped by different clinicians using different levels of resolution (with/without electron microscopy), genetic diagnoses were observed in both cohorts in comparable numbers, with five out of nine Argentinian patients genetically diagnosed versus 5 out of 12 Australian patients. Interestingly, however, exome sequencing revealed variants in either known or candidate genes in all Argentinean patients but not in all Australian patients, leaving four Australian patients without mutations in known or novel candidate genes. The reason for this difference is not currently known but is likely related to the differences in the population background between the two locations.

Patient ARG3 carried two likely pathogenic variants (c.1316 + 1_1316 + 2ins (canonical splice site variant) and c.7762C>T; p.(Arg2588*)) in *DNAH6* and no variants in other candidate genes (Table III). *DNAH6* is a dynein gene involved in motile cilia function in numerous tissues, which, when mutated, leads to a primary cilia dyskinesia phenotype in zebra fish and humans (Li *et al*., 2016). DNAH6 has also been recently implicated in the aetiology of human DFS-MMAF and confirmed in a mouse study (Tu *et al*., 2019). Herein we confirm that mutations in *DNAH6* are a bona fide cause of human DFS-MMAF and that DFS-MMAF should be considered as part of the spectrum of clinical presentations designated as severe sperm motility syndrome.

In two other cases, we are not convinced that the sperm motility defects can be explained by variants in *DNAH6*. The two *DNAH6* variants in AUS3 (c.9436A>G; p.(Ser3146Gly) and c.12352G>A; p.(Ala4118Thr)) (Supplementary Table SIII) have almost identical minor allele frequencies among the gnomAD populations, indicating they are located on the same allele and are not compound heterozygous. This hypothesis could not be tested due to the unavailability of parental DNA of this patient, but this makes it unlikely that these variants alone cause DFS-MMAF in AUS3. The other patient with a candidate variant in *DNAH6* is ARG8, carrying a homozygous variant of unknown significance (VUS) (c.2059C>A; p.(Pro687Thr)). This patient, however, also carried variants of unclear significance in three other genes: (1) *ATP2B4,* which is associated with asthenozoospermia in mice; (2) *CEP290*, mutations which are a known cause of Leber’s Congenital Amaurosis that is associated with asthenozoospermia in males; and (3) *CEP350*, which is known to interact with *CEP290* and the known MMAF gene *CEP135*. It remains uncertain whether variants in any of these genes alone, or combined, are responsible for DFS-MMAF in combination with chronic bronchitis.

The variant (p.(Gln454*)) identified in *CFAP58* in patient ARG5 has a low allele frequency in gnomAD (0.054%), but appears to be more common among the Ashkenazi Jew (ASJ) population reported in the same database (1.086%). This means that an expected 0.012% of this population is homozygous for this variant; a number that is slightly higher than the estimated frequency of DFS-MMAF in the population of Dutch men (0.005–0.01%) (our own observations). Although the variant identified in ARG5 almost certainly disrupts CFAP58 protein function, it remains unclear if this variant is underlying the DFS-MMAF phenotype in the patient due to the allele frequency, which is higher than expected in the ASJ population and additional population studies are required. This variant was located in a homozygosity stretch, indicating consanguinity. The brother of this patient also presented with DFS-MMAF, but DNA was unavailable.

Interestingly, the semen analysis of ARG6, who carried a homozygous frameshift variant (p.(Arg218Aspfs*37)) in *CFAP44,* showed the combination of DFS-MMAF and acephalic sperm previously reported in the literature (Rawe *et al*., 2002; Moretti *et al*., 2011). This indicates the possibility of combinations between different sperm phenotypes of genetic origin or involvement of a single gene/protein associated with transport pathways common between the sperm head-tail coupling apparatus and tail proteins (reviewed by Pleuger *et al*., 2020).

### Novel candidate genes for severe sperm motility disorders

Since variants in known genes explain causality in approximately half of our patients, we investigated whether genetic variants were present in genes with a potential role in sperm function. In the current study, we observed missense and null mutations in six novel genes (*DNAH12*, *PACRG*, *DRC1*, *MDC1*, *SSPL2C* and *TPTE2*) that have previously been identified to play a role in axoneme assembly and/or sperm flagellum development and have been shown to interact with genes already implicated in sperm function (Pleuger *et al*., 2020; Toure *et al*., 2020).

Patient ARG1 carried two missense variants (p.(Phe1298Ser) and p.(Pro2480Ser)) in *DNAH12,* which is the closest paralog of the *DNAH1* gene. Pathogenic variants in *DNAH1* are known to cause classical PCD or DFS-MMAF without any PCD symptoms (Ben Khelifa *et al*., 2014; Wambergue *et al*., 2016; Wang *et al*., 2017; Amiri-Yekta *et al*., 2016; Sha *et al*., 2017; Coutton *et al*., 2018; Sha *et al*., 2019b; Coutton *et al*., 2019; Li *et al*., 2019b; Hu *et al*., 2019). The allele frequencies of both *DNAH12* variants are similar in three different populations in gnomAD, which could indicate they reside on the same allele. It is therefore unclear whether these variants are indeed bi-allelic and causal of infertility. Another patient, ARG4, carried a homozygous nonsense variant (p.(Tyr123*)) in *PACRG.* This gene has been implicated in motile cilia function and mutations in mice are known to cause male infertility characterised by defective sperm head and tail formation in combination with hydrocephalus next to fertility problems (Lorenzetti *et al.*, 2004; Wilson *et al*., 2010; Li *et al*., 2015). The variants identified in ARG1 and ARG4 likely explain the DFS-MMAF phenotype seen in both patients.

Patient ARG7 carried two nonsense variants (p.(Arg80*) and p.(Gln118*)) in *DRC1*. This gene is known to be important for motile cilia formation, and specifically outer dynein arm formation, as concluded from studies in algae (Wirschell *et al*., 2013). Loss-of-function point mutations and a recurrent ∼28 kb deletion encompassing *DRC1* Exons 1–4 have previously been described in patients with PCD, including a man who had undergone fertility treatment (Wirschell *et al*., 2013; Morimoto *et al*., 2019a). Unfortunately, sperm ultrastructure was not examined. The second nonsense variant (p.(Gln118*)) has been described in two Swedish PCD families (Carlen *et al*., 2003; Wirschell *et al*., 2013). The importance of *DRC1* in human spermatogenesis is further strengthened by the observed enhanced expression in the testis (along with the brain and fallopian tube) (Uhlen *et al*., 2010; GTEx Consortium, 2015). Collectively, these data suggest that mutations in *DRC1* cause a spectrum of clinical presentations involving defects in motile cilia function, and that variants in *DRC1* are a novel high confidence cause of male infertility.

In addition to variants in genes with a strong link the clinical aetiology of DFS-MMAF, we also identified variants in genes with less well characterised links to sperm motility. First, patient AUS4 carried a homozygous missense variant of unknown significance (p.(Arg212Trp)) in *SPPL2C*. This gene encodes a testis-specific intermembrane protease residing in the endoplasmic reticulum in somatic cells and in elongating spermatids in the testis (Niemeyer *et al*., 2019; Papadopoulou *et al*., 2019). In mice, *Sppl2c* deficiency leads to hypospermatogenesis starting at the level of spermatids, as well as reduced sperm motility and male sub-fertility (Niemeyer *et al*., 2019). The effect of *Sppl2c* deletion on the sperm ultra-structure was not examined and, as such, a definitive link to DFS-MMAF cannot be made. *SPPL2C* is also one of multiple genes deleted in Koolen–de Vries 17q21.31 microdeletion syndrome (Koolen *et al*., 2006; Shaw-Smith *et al*., 2006). A recent case report of Koolen–de Vries syndrome described a patient with intellectual disability and oligoastheno-teratozoospermia. Although it is unlikely that disruption of *SPPL2C* has an effect on intellectual disability, it is possible that its disruption is causative for the infertility described in this patient.

Patient AUS7 carried a homozygous nonsense variant (c.715C>T; p.(Gln239*)) in the voltage-sensitive and membrane-associated phosphatase *TPTE2*. The expression of this gene is highly testis enriched and the protein is localised within the sperm plasma membrane, where it is likely involved in integrating environmental cues into changes in sperm function (Sutton *et al*., 2012). The homozygous nonsense variant is located in exon 8 and likely results in nonsense-mediated mRNA decay. Based on these data, while we predict mutations in *TPTE2* are likely to result in male infertility, a specific link to the mechanisms underpinning sperm tail assembly is lacking. As such, we classify mutations in *TPTE2* as a possible, but not high confidence cause of severe sperm motility disorders.

Lastly, in patient AUS11, we identified two heterozygous nonsense variants in *MDC1*, a gene essential for the silencing of sex chromosome and genome stability during male meiosis in mice (Ichijima *et al*., 2011). Unfortunately, parental DNA was not available to prove the biallelic presence of these two heterozygous variants. The knockout mouse model of *Mdc1* revealed a meiotic arrest (Lou *et al*., 2006), a phenotype that does not directly match the phenotype seen in AUS11. The semen analysis of AUS11 revealed moderate oligozoospermia (8.8 million sperm/ml) and only 5% of sperm showed progressive motility. Based on currently available information on *MDC1*, while we are confident mutations in *MDC1* can lead to human male infertility, they are not a high confidence cause of the severe motility disorder seen in this patient.

### Comparison of our results to exome sequencing in a control cohort

The vast majority of known variants causing sperm tail assembly disorders, are homozygous LoF variants (Supplementary Table SI; Oud *et al*., 2019). Sequencing of a control cohort of 5784 proven fathers did not reveal a similarly high number of homozygous LoF variants in sperm motility genes (Supplementary Table SV). This shows that these disruptive variants occur only rarely in the normal male population, in contrast to males presenting with severe sperm motility disorders. These results further strengthen the evidence for the involvement of these genes in abnormal sperm tail assembly.

Interestingly, we did identify one homozygous LoF variant in a proven father in *CFAP69,* a recently discovered gene which is strongly associated with the DFS-MMAF phenotype (Dong *et al*., 2018; He *et al*., 2019). Although it is possible that this man had ICSI to conceive his child, it does indicate that homozygous knock-out of this gene may not always cause complete sterility in human. Due to data usage restrictions, we are unable to search for compound heterozygous variants and could only investigate the zygosity and frequency of all variants in the entire cohort.

### Importance for genetic testing in severe sperm motility disorders

Both the European and American guidelines for genetic testing in male infertility, provide a stratified approach to select azoospermic and oligozoospermic patients based on clinical phenotypes to certain genetic tests (Esteves and Chan, 2015; Jungwirth, 2018). The guidelines, however, do not include recommendations for patients with other sperm phenotypes including severe sperm motility disorders. Without a genetic diagnosis, a clinician is very restricted to accurately counsel couples with questions about the causes of their infertility, possible co-morbidities, the potential success of ART treatment and the (reproductive) health of their offspring. Hence, understanding of and testing for genetic causes of severe sperm motility disorders are of enormous value to patients and clinicians. Currently, it remains unclear if genetic abnormalities underlying sperm motility disorders affect the health of potential offspring. As such, the field should consider expanding diagnostic genetic testing for this group of patients, especially since this and other recent studies have reported high (>40%) diagnostic yields in these patient groups (Toure *et al*., 2020). Furthermore, systematic linking of genetic data with ART success rates as well as patient and offspring health is pivotal for improved counselling in this group of patients.

## Conclusion

In summary, our genetic data provided a diagnosis for 10 out of 21 patients with severe sperm motility disorders and we discovered novel candidate genes in seven other patients. Functional data based on literature, propose variants in *DNAH12, DRC1, MDC1, PACRG, SPPL2C* and *TPTE2* as novel genetic causes of severe sperm motility disorders. Our results demonstrate that exome-wide screening for pathogenic variants in these genes is an effective way to diagnose severe forms of motility disorders (Supplementary Table SI). Exome sequencing of additional cases and re-analysis of exome data of currently unsolved cases from other cohorts may reveal additional causative mutations in these novel candidate genes.

## Data availability

Raw and processed data are available under controlled access and requires a Data Transfer Agreement from the European Genome-Phenome Archive (EGA) repository: EGAS00001005018. Data is available using the following link: https://ega-archive.org/studies/EGAS00001005018

## Supplementary Material

deab099_Supplementary_TableS1

deab099_Supplementary_TableS2

deab099_Supplementary_TableS3

deab099_Supplementary_TableS4

deab099_Supplementary_TableS5
