# Supplementary material for: Exome sequencing reveals variants in known and novel candidate genes for severe sperm motility disorders
Source: Hum Reprod. 2021 Jun 5;36(9):2597–611. doi: 10.1093/humrep/deab099 (PMC8373475; doi:10.1093/humrep/deab099)
Supplement: deab099_Supplementary_TableS1 [file deab099_supplementary_tables1.pdf]

Human Reproduction, pp. 1–5, 2021
doi:10.1093/humrep/deab099

human reproduction

SUPPLEMENTARY DATA

Supplementary Table SI **Previously described and novel candidate genes for DFS-MMAF and related sperm phenotypes.**

Gene symbol	Synonym	Full gene name	Proposed protein function	Variants in humans	Types of variants described	Absence of homozygous loss of function variation in proven fathers?	Reference
AK7		Adenylate Kinase 7	Phosphotransferase involved in the interconversion of adenine nucleotides. Potentially involved in maintaining motile cilia axoneme stability. Loss of function leads to a spectrum of conditions ranging from pure PCD to severe teratozospermia characterised by poor axoneme elongation and reduced sperm output.	Previously described	Missense	Yes	Lores *et al.* (2018), Fernandez-Gonzalez *et al.* (2009) and Panayiotou *et al.* (2011)
AKAP4		A-Kinase Anchoring Protein 4	Scaffold protein for cAMP-dependent protein kinases and thereby localise sites for protein phosphorylation. Major component of the sperm fibrous sheath. Loss of protein function leads to fibrous sheath disorganisation and compromised glycolysis and thus sperm motility.	Previously described	Missense	Yes	Visser *et al.* (2011) and Miki *et al.* (2002)
ARMC2		Armadillo Repeat Containing 2	Potential involvement in the assembly or stability of the axoneme central microtubule pair. As yet unknown biochemical function. Defects in axoneme and sperm tail accessory structure assembly. Oligozoospermia.	Previously described	LoF and missense	Yes	Coutton *et al.* (2019)
CEP135		Centrosomal Protein 135	A coiled coil domain containing protein involved in microtubule bunding and centriole assembly and stability.	Previously described	Missense	Yes	Sha *et al.* (2017) and Kraatz *et al.* (2016)
CFAP43	*WDR96*	Cilia And Flagella Associated Protein 43	As yet unknown biochemical function. Proposed role in centriole function and axoneme extension from the centriole. Disfunction leads to defects in motile cilia function including hydrocephalus, grossly disorganised sperm axoneme structure and length and associated sperm tail accessory structure defects.	Previously described and described in this study	LoF and missense	Yes	Tang *et al.* (2017), McLachlan and Krausz (2012), Coutton *et al.* (2018), Sha *et al.* (2019b), Coutton *et al.* (2019), Li *et al.* (2019b), Wu *et al.* (2019), Morimoto *et al.* (2019b)<br>This study
CFAP44	*WDR52*	Cilia And Flagella Associated Protein 44	As yet unknown biochemical function. Proposed role in centriole function, axoneme extension and/or stability. Dysfunction leads to grossly disorganised sperm axoneme structure and length and associated sperm tail accessory structure defects.	Previously described	LoF and missense	Yes	Tang *et al.* (2017), Escudier et al. (2009), Coutton *et al.* (2018), Sha *et al.* (2019b), Coutton *et al.* (2019), Li *et al.* (2019b) and Wu *et al.* (2019)<br>This study
CFAP58					LoF	Yes	He *et al.* (2020)

(continued)

Human Reproduction, pp. 17–24, 2021
doi:10.1093/humrep/deab099

Supplementary Table SI Continued

Gene symbol	Synonym	Full gene name	Proposed protein function	Variants in humans	Types of variants described	Absence of homozygous loss of function variation in proven fathers?	Reference
		Cilia And Flagella Associated Protein 58	Unknown biochemical function. Localised to motile cilia in the fallopian tube. No available animal models.	Novel candidate gene described in this study			This study
CFAP65	*CCDC108*	Cilia And Flagella Associated Protein 65	Biochemical function unknown. Proposed role in axoneme extension and/or stability.<br>Defects in humans are associated with PCD-like phenotype including respiratory tract disease and MMAF in spermatozoa plus acrosome defects.	Previously described	LoF and missense	Yes	Wang *et al.* (2019), Li *et al.* (2019a), and Zhang *et al.* (2019b)
CFAP69		Cilia And Flagella Associated Protein 69	Biochemical function unknown. Proposed role in axoneme extension/stability and sperm tail organisation. Localised to the mid-piece of normal sperm.<br>Dysfunction leads to PCD symptoms including respiratory disease and MMAF in spermatozoa including the absence of axoneme central microtubule pair.	Previously described	LoF	No, 1/5784 proven fathers carried a homozygous Chr7(GRCh37):g.89906394del; NM_001039706.2(CFAP69):c.992del; p.(Gly331Alafs*6) variant	Dong et al. (2018) and He *et al.* (2019)
CFAP70	*TTC18*	Cilia And Flagella Associated Protein 70	Biochemical function is unknown. Proposed function in protein transport.<br>Associated with the axoneme in motile cilia. Dysfunction of the orthologous protein in trypanosomes leads to the loss of outer dynein arms.	Previously described	LoF and missense	Yes	Beurois *et al.* (2019) and Shamoto *et al.* (2018)
DNAH1		Dynein Axonemal Heavy Chain 1	Inner dynein arm-associated dynein heavy chain. Proposed additional role in axoneme assembly or stability.<br>Dysfunction results in PCD symptoms. In the mouse loss of function leads to sterility associated with sperm clumping and compromised motility.	Previously described	LoF and missense	Yes	Ben Khelifa *et al.* (2014), Wambergue *et al.* (2016), Wang *et al.* (2017), Amiri-Yekta *et al.* (2016), Sha *et al.* (2017), Coutton *et al.* (2018), Sha *et al.* (2019b), Coutton *et al.* (2019), Li *et al.* (2019b), and Hu *et al.* (2019)<br>This study
DNAH2		Dynein Axonemal Heavy Chain 2	Axonemal inner arm dynein heavy chain protein. Proposed additional role in axoneme assembly or stability.	Previously described	LoF and missense	Yes	Li *et al.* (2019b)
DNAH6		Dynein Axonemal Heavy Chain 6	Axonemal dynein heavy chain protein. Proposed role in axoneme assembly or stability. Highly expressed in tissues	Previously described and	LoF and missense	Yes	Tu *et al.* (2019) and Gershoni *et al.* (2017)<br>This study

(continued)

Human Reproduction, pp. 17–24, 2021
doi:10.1093/humrep/deab099

Supplementary Table SI Continued

Gene symbol	Synonym	Full gene name	Proposed protein function	Variants in humans	Types of variants described	Absence of homozygous loss of function variation in proven fathers?	Reference
			containing motile cilia and associated with known PCD genes. Evidence of respiratory disease in some patients.	described in this study			
DNAH12		Dynein Axonemal Heavy Chain 12	Dynein heavy chain protein. Its function has not been tested experimentally.	Novel candidate gene described in this study	Unknown	Yes	This study
DNAH17		Dynein Axonemal Heavy Chain 17	Dynein outer dynein arm heavy chain protein. Proposed role in axoneme stability. Its function has not been tested experimentally.	Previously described	LoF and missense	Yes	Whitfield et al. (2019), Zhang *et al.* (2019a), and Sha *et al.* (2019a)
DRC1	*CCDC164*	Dynein Regulatory Complex Subunit 1	A component of the nexin-dynein regulatory complex involved in the regulation of dynein motors and coordination between the central microtubule pair and the outer dyein arms within the axoneme. Mutations are associated with PCD symptoms including sinopulmonary disease—male fertility was not explored.	Novel candidate gene described in this study	Unknown	Yes	Keicho *et al.* (2020), Morimoto *et al.* (2019a), Wirschell *et al.* (2013) This study
DZIP1		DAZ Interacting Zinc Finger Protein 1	Associated with centrosomes and ciliogenesis	Previously described	LoF and missense	Yes	Lv *et al.* (2020)
FSIP2		Fibrous Sheath Interacting Protein 2	AKAP4 binding protein of unknown biochemical function. Fibrous sheath localisation. Proposed role in fibrous sheath assembly and sperm tail stability.	Previously described	LoF	Yes	Martinez *et al.* (2018), Liu *et al.* (2019e), Coutton *et al.* (2019), and Brown *et al.* (2003)
MAATS1	*CFAP91*	MYCBP Associated And Testis Expressed 1	Axonemal protein with a likely function in the central pair complex	Previously described	LoF and missense	Yes	Martinez *et al.* (2020)
MDC1		Mediator Of DNA Damage Checkpoint 1	Silencing of sex chromosome and genome stability	Novel candidate gene described in this study	Unknown	Yes	This study
PACRG		Parkin Coregulated	Key component of the junction between the A- And B-tubules within the axoneme and thus ciliary dyein function. Potential role in protein transport into the developing sperm tail.	Novel candidate gene described in this study	Unknown	Yes	This study

(continued)

Human Reproduction, pp. 17–24, 2021
doi:10.1093/humrep/deab099

Supplementary Table SI Continued

Gene symbol	Synonym	Full gene name	Proposed protein function	Variants in humans	Types of variants described	Absence of homozygous loss of function variation in proven fathers?	Reference
			Dysfunction leads to compromised inner dynein arm assembly and is associated with PCD including hydrocephalus.				
QRICH2		Glutamine Rich 2	Undefined biochemical function. A potential role in intra-flagella protein transport. Dysfunction leads to sperm tail malformation and instability.	Previously described and described in this study	LoF and missense	Yes	Shen *et al.* (2019) and Kherraf *et al.* (2019) This study
SPEF2		Sperm Flagellar 2	IFT20 interacting protein with a proposed role in intra-manchette protein transport. Dysfunction leads to PCD in humans. Involved in manchette migration, and thus sperm head shaping, in mouse spermatogenesis. Also involved in regulating centriole duplication and formation/stability of the axoneme central microtubule pair.	Previously described	LoF	Yes	Liu *et al.* (2019b), Liu *et al.* (2019d), Sha *et al.* (2019a), and Tu *et al.* (2020)
SPPL2C		Signal Peptide Peptidase Like 2C	A catalytically active GxGD-type intramembrane protease involved in vesicle transport. Proposed role in acrosome formation. Function untested in the context of spermatogenesis.	Novel candidate gene described in this study	Unknown	Yes	This study
TPTE2		Transmembrane Phosphoinositide 3-Phosphatase And Tensin Homolog 2	Transmembrane voltage sensor. Untested role in male fertility.	Novel candidate gene described in this study	Unknown	Yes	This study
TTC29		Tetratricopeptide Repeat Domain 29	Required for delivery of key proteins into the sperm tail via intra-flagellar transport pathway. The loss of *TTC29* in the mouse leads to severely disrupted axoneme and accessory structure formation. The precise biochemical function is unknown.	Previously described	LoF	Yes	Liu *et al.* (2019a) and Lores *et al.* (2019)
TTC21A	*IFT139A*	Tetratricopeptide Repeat Domain 21 A	Proposed role in intra-flagellar transport and sperm tail stability. Dysfunction in humans and mice leads to sperm tail disorganisation including missing central axoneme microtubules, sperm neck/connecting piece defects and short tails.	Previously described	LoF and missense	Yes	Liu et al. (2019c)

(continued)

Human Reproduction, pp. 17–24, 2021
doi:10.1093/humrep/deab099

Supplementary Table SI Continued

Gene symbol	Synonym	Full gene name	Proposed protein function	Variants in humans	Types of variants described	Absence of homozygous loss of function variation in proven fathers?	Reference
WDR66	*CFAP251*	WD Repeat Domain 66	An axoneme associated protein with a proposed role in axoneme central pair formation and sperm tail stability. Loss of function lead to sperm tail disorganisation and shortening. Also observed acrosomal defects. Loss of function of the orthologous protein in T.brucei resulted in compromised flagella motility characterised by axoneme disorganisation. The central microtubule pair of the axoneme were notably affected.	Previously described	LoF	Yes	Kherraf et *al.* (2018), Auguste et *al.* (2018), Li et *al.* (2019a), and Coutton et *al.* (2019)

LoF, loss-of-function (nonsense, canonical splice site and frameshift variation).

References

Amiri-Yekta A, Coutton C, Kherraf ZE, Karaouzene T, Le Tanno P, Sanati MH, Sabbaghian M, Almadani N, Sadighi Gilani MA, Hosseini SH. *et al.* Whole-exome sequencing of familial cases of multiple morphological abnormalities of the sperm flagella (MMAF) reveals new DNAH1 mutations. *Hum Reprod* 2016;**31**:2872–2880.

Auguste Y, Delague V, Desvignes JP, Longepied G, Gnisci A, Besnier P, Levy N, Beroud C, Megarbane A, Metzler-Guillemain C. *et al.* Loss of calmodulin- and radial-spoke-associated complex protein CFAP251 leads to immotile spermatozoa lacking mitochondria and infertility in men. *Am J Hum Genet* 2018;**103**:413–420.

Ben Khelifa M, Coutton C, Zouari R, Karaouzene T, Rendu J, Bidart M, Yassine S, Pierre V, Delaroche J, Hennebicq S. *et al.* Mutations in DNAH1, which encodes an inner arm heavy chain dynein, lead to male infertility from multiple morphological abnormalities of the sperm flagella. *Am J Hum Genet* 2014;**94**:95–104.

Beurois J, Martinez G, Cazin C, Kherraf ZE, Amiri-Yekta A, Thierry-Mieg N, Bidart M, Petre G, Satre V, Brouillet S. *et al.* CFAP70 mutations lead to male infertility due to severe astheno-teratozoospermia. A case report. *Hum Reprod* 2019;**34**:2071–2079.

Brown PR, Miki K, Harper DB, Eddy EM. A-kinase anchoring protein 4 binding proteins in the fibrous sheath of the sperm flagellum. *Biol Reprod* 2003;**68**:2241–2248.

Carlen B, Lindberg S, Stenram U. Absence of nexin links as a possible cause of primary ciliary dyskinesia. *Ultrastruct Pathol* 2003;**27**: 123–126.

Coutton C, Martinez G, Kherraf ZE, Amiri-Yekta A, Boguenet M, Saut A, He X, Zhang F, Cristou-Kent M, Escoffier J. *et al.* Bi-allelic mutations in ARMC2 lead to severe astheno-teratozoospermia due to sperm flagellum malformations in humans and mice. *Am J Hum Genet* 2019;**104**:331–340.

Coutton C, Vargas AS, Amiri-Yekta A, Kherraf ZE, Ben Mustapha SF, Le Tanno P, Wambergue-Legrand C, Karaouzene T, Martinez G, Crouzy S. *et al.* Mutations in CFAP43 and CFAP44 cause male infertility and flagellum defects in Trypanosoma and human. *Nat Commun* 2018;**9**:686.

Dong FN, Amiri-Yekta A, Martinez G, Saut A, Tek J, Stouvenel L, Lores P, Karaouzene T, Thierry-Mieg N, Satre V. *et al.* Absence of CFAP69 causes male infertility due to multiple morphological abnormalities of the flagella in human and mouse. *Am J Hum Genet* 2018;**102**:636–648.

Dumur V, Gervais R, Rigot JM, Lafitte JJ, Manouvrier S, Biserte J, Mazeman E, Roussel P. Abnormal distribution of CF delta F508 allele in azoospermic men with congenital aplasia of epididymis and vas deferens. *Lancet* 1990;**336**:512.

Escudier E, Duquesnoy P, Papon JF, Amselem S. Ciliary defects and genetics of primary ciliary dyskinesia. *Paediatr Respir Rev* 2009;**10**:51–54.

Esteves SC, Chan P. A systematic review of recent clinical practice guidelines and best practice statements for the evaluation of the infertile male. *Int Urol Nephrol* 2015;**47**:1441–1456.

Human Reproduction, pp. 17–24, 2021
doi:10.1093/humrep/deab099

human reproduction

SUPPLEMENTARY DATA

Fernandez-Gonzalez A, Kourembanas S, Wyatt TA, Mitsialis SA. Mutation of murine adenylate kinase 7 underlies a primary ciliary dyskinesia phenotype. *Am J Respir Cell Mol Biol* 2009;**40**:305–313.

Gershoni M, Hauser R, Yogev L, Lehavi O, Azem F, Yavetz H, Pietrokovski S, Kleiman SE. A familial study of azoospermic men identifies three novel causative mutations in three new human azoospermia genes. *Genet Med* 2017;**19**:998–1006.

Halaszovich CR, Leitner MG, Mavrantoni A, Le A, Frezza L, Feuer A, Schreiber DN, Villalba-Galea CA, Oliver D. A human phospholipid phosphatase activated by a transmembrane control module. *J Lipid Res* 2012;**53**:2266–2274.

He X, Li W, Wu H, Lv M, Liu W, Liu C, Zhu F, Li C, Fang Y, Yang C. *et al.* Novel homozygous CFAP69 mutations in humans and mice cause severe asthenoteratospermia with multiple morphological abnormalities of the sperm flagella. *J Med Genet* 2019;**56**:96–103.

He X, Liu C, Yang X, Lv M, Ni X, Li Q, Cheng H, Liu W, Tian S, Wu H. *et al.* Bi-allelic loss-of-function variants in CFAP58 cause flagellar axoneme and mitochondrial sheath defects and asthenoteratozoospermia in humans and mice. *Am J Hum Genet* 2020;**107**:514–526.

Houston BJ, Oud MS, Aguirre DM, Merriner DJ, O'Connor AE, Okutman O, Viville S, Burke R, Veltman JA, O'Bryan MK. Programmed cell death 2-like (Pdcd2l) is required for mouse embryonic development. *G3 (Bethesda)* 2020;**7**:247–255.

Hu J, Lessard C, Longstaff C, O'Brien M, Palmer K, Reinholdt L, Eppig J, Schimenti J, Handel MA. ENU-induced mutant allele of Dnah1, ferf1, causes abnormal sperm behavior and fertilization failure in mice. *Mol Reprod Dev* 2019;**86**:416–425.

Ichijima Y, Ichijima M, Lou Z, Nussenzweig A, Camerini-Otero RD, Chen J, Andreassen PR, Namekawa SH. MDC1 directs chromosome-wide silencing of the sex chromosomes in male germ cells. *Genes Dev* 2011;**25**:959–971.

Keicho N, Hijikata M, Morimoto K, Homma S, Taguchi Y, Azuma A, Kudoh S. Primary ciliary dyskinesia caused by a large homozygous deletion including exons 1–4 of DRC1 in Japanese patients with recurrent sinopulmonary infection. *Mol Genet Genomic Med* 2020; **8**:e1033.

Kherraf ZE, Amiri-Yekta A, Dacheux D, Karaouzene T, Coutton C, Christou-Kent M, Martinez G, Landrein N, Le Tanno P, Fourati Ben Mustapha S. *et al.* A homozygous ancestral SVA-insertion-mediated deletion in WDR66 induces multiple morphological abnormalities of the sperm flagellum and male infertility. *Am J Hum Genet* 2018;**103**:400–412.

Kherraf ZE, Cazin C, Coutton C, Amiri-Yekta A, Martinez G, Boguenet M, Fourati Ben Mustapha S, Kharouf M, Gourabi H, Hosseini SH. *et al.* Whole exome sequencing of men with multiple morphological abnormalities of the sperm flagella reveals novel homozygous QRICH2 mutations. *Clin Genet* 2019;**96**:394–401.

Kraatz S, Guichard P, Obbineni JM, Olieric N, Hatzopoulos GN, Hilbert M, Sen I, Missimer J, Gonczy P, Steinmetz MO. The human centriolar protein CEP135 contains a two-stranded coiled-coil domain critical for microtubule binding. *Structure* 2016;**24**:1358–1371.

Lancaster MA, Gopal DJ, Kim J, Saleem SN, Silhavy JL, Louie CM, Thacker BE, Williams Y, Zaki MS, Gleeson JG. Defective Wnt-dependent cerebellar midline fusion in a mouse model of Joubert syndrome. *Nat Med* 2011;**17**:726–731.

Li W, He X, Yang S, Liu C, Wu H, Liu W, Lv M, Tang D, Tan J, Tang S. *et al.* Biallelic mutations of CFAP251 cause sperm flagellar defects and human male infertility. *J Hum Genet* 2019a;**64**:49–54.

Li W, Tang W, Teves ME, Zhang Z, Zhang L, Li H, Archer KJ, Peterson DL, Williams DC Jr, Strauss JF. *et al.* A MEIG1/PACRG complex in the manchette is essential for building the sperm flagella. *Development* 2015;**142**:921–930.

Li W, Wu H, Li F, Tian S, Kherraf ZE, Zhang J, Ni X, Lv M, Liu C, Tan Q. *et al.* Biallelic mutations in CFAP65 cause male infertility with multiple morphological abnormalities of the sperm flagella in humans and mice. *J Med Genet* 2019b;**57**;89–95.

Li Y, Sha Y, Wang X, Ding L, Liu W, Ji Z, Mei L, Huang X, Lin S, Kong S. *et al.* DNAH2 is a novel candidate gene associated with multiple morphological abnormalities of the sperm flagella. *Clin Genet* 2019c;**95**:590–600.

Li Y, Yagi H, Onuoha EO, Damerla RR, Francis R, Furutani Y, Tariq M, King SM, Hendricks G, Cui C. *et al.* DNAH6 and its interactions with PCD genes in heterotaxy and primary ciliary dyskinesia. *PLoS Genet* 2016;**12**:e1005821.

Liu C, He X, Liu W, Yang S, Wang L, Li W, Wu H, Tang S, Ni X, Wang J. *et al.* Bi-allelic mutations in TTC29 cause male subfertility with asthenoteratospermia in humans and mice. *Am J Hum Genet* 2019a;**105**:1168–1181.

Liu C, Lv M, He X, Zhu Y, Amiri-Yekta A, Li W, Wu H, Kherraf ZE, Liu W, Zhang J. *et al.* Homozygous mutations in SPEF2 induce multiple morphological abnormalities of the sperm flagella and male infertility. *J Med Genet* 2019b;**57**:31–37.

Liu W, He X, Yang S, Zouari R, Wang J, Wu H, Kherraf ZE, Liu C, Coutton C, Zhao R. *et al.* Bi-allelic mutations in TTC21A induce asthenoteratospermia in humans and mice. *Am J Hum Genet* 2019c;**104**:738–748.

Liu W, Sha Y, Li Y, Mei L, Lin S, Huang X, Lu J, Ding L, Kong S, Lu Z. Loss-of-function mutations in SPEF2 cause multiple morphological abnormalities of the sperm flagella (MMAF). *J Med Genet* 2019d;**56**:678–684.

Liu W, Wu H, Wang L, Yang X, Liu C, He X, Li W, Wang J, Chen Y, Wang H. *et al.* Homozygous loss-of-function mutations in FSIP2 cause male infertility with asthenoteratospermia. *J Genet Genomics* 2019e;**46**:53–56.

Lorenzetti D, Bishop CE, Justice MJ. Deletion of the Parkin coregulated gene causes male sterility in the quaking(viable) mouse mutant. *Proc Natl Acad Sci U S A* 2004;**101**:8402–8407.

Lores P, Coutton C, El Khouri E, Stouvenel L, Givelet M, Thomas L, Rode B, Schmitt A, Louis B, Sakheli Z. *et al.* Homozygous missense mutation L673P in adenylate kinase 7 (AK7) leads to primary male infertility and multiple morphological anomalies of the flagella but not to primary ciliary dyskinesia. *Hum Mol Genet* 2018;**27**:1196–1211.

Human Reproduction, pp. 17–24, 2021
doi:10.1093/humrep/deab099

Lores P, Dacheux D, Kherraf ZE, Nsota Mbango JF, Coutton C, Stouvenel L, Ialy-Radio C, Amiri-Yekta A, Whitfield M, Schmitt A. *et al.* Mutations in TTC29, encoding an evolutionarily conserved axonemal protein, result in asthenozoospermia and male infertility. *Am J Hum Genet* 2019;**105**:1148–1167.

Lou Z, Minter-Dykhouse K, Franco S, Gostissa M, Rivera MA, Celeste A, Manis JP, van Deursen J, Nussenzweig A, Paull TT. *et al.* MDC1 maintains genomic stability by participating in the amplification of ATM-dependent DNA damage signals. *Mol Cell* 2006; **21**:187–200.

Lv M, Liu W, Chi W, Ni X, Wang J, Cheng H, Li WY, Yang S, Wu H, Zhang J. *et al.* Homozygous mutations in DZIP1 can induce asthenoteratospermia with severe MMAF. *J Med Genet* 2020;**57**: 445–453.

Martinez G, Beurois J, Dacheux D, Cazin C, Bidart M, Kherraf ZE, Robinson DR, Satre V, Le Gac G, Ka C. *et al.* Biallelic variants in MAATS1 encoding CFAP91, a calmodulin-associated and spoke-associated complex protein, cause severe astheno-teratozoospermia and male infertility. *J Med Genet* 2020;**0**:1–9.

Martinez G, Kherraf ZE, Zouari R, Fourati Ben Mustapha S, Saut A, Pernet-Gallay K, Bertrand A, Bidart M, Hograindleur JP, Amiri-Yekta A. *et al.* Whole-exome sequencing identifies mutations in FSIP2 as a recurrent cause of multiple morphological abnormalities of the sperm flagella. *Hum Reprod* 2018;**33**:1973–1984.

Miki K, Willis WD, Brown PR, Goulding EH, Fulcher KD, Eddy EM. Targeted disruption of the Akap4 gene causes defects in sperm flagellum and motility. *Dev Biol* 2002;**248**:331–342.

Moretti E, Geminiani M, Terzuoli G, Renieri T, Pascarelli N, Collodel G. Two cases of sperm immotility: a mosaic of flagellar alterations related to dysplasia of the fibrous sheath and abnormalities of head-neck attachment. *Fertil Steril* 2011;**95**;1787. e1719–1723.

Morimoto K, Hijikata M, Zariwala MA, Nykamp K, Inaba A, Guo TC, Yamada H, Truty R, Sasaki Y, Ohta K. *et al.* Recurring large deletion in DRC1 (CCDC164) identified as causing primary ciliary dyskinesia in two Asian patients. *Mol Genet Genomic Med* 2019a;**7**: e838.

Morimoto Y, Yoshida S, Kinoshita A, Satoh C, Mishima H, Yamaguchi N, Matsuda K, Sakaguchi M, Tanaka T, Komohara Y. *et al.* Nonsense mutation in CFAP43 causes normal-pressure hydrocephalus with ciliary abnormalities. *Neurology* 2019b;**92**: e2364–e2374.

Neesen J, Kirschner R, Ochs M, Schmiedl A, Habermann B, Mueller C, Holstein AF, Nuesslein T, Adham I, Engel W. Disruption of an inner arm dynein heavy chain gene results in asthenozoospermia and reduced ciliary beat frequency. *Hum Mol Genet* 2001;**10**: 1117–1128.

Niemeyer J, Mentrup T, Heidasch R, Muller SA, Biswas U, Meyer R, Papadopoulou AA, Dederer V, Haug-Kroper M, Adamski V. *et al.* The intramembrane protease SPPL2c promotes male germ cell development by cleaving phospholamban. *EMBO Rep* 2019;**20**: e46449.

Panayiotou C, Solaroli N, Xu Y, Johansson M, Karlsson A. The characterization of human adenylate kinases 7 and 8 demonstrates differences in kinetic parameters and structural organization among the family of adenylate kinase isoenzymes. *Biochem J* 2011;**433**: 527–534.

Papadopoulou AA, Muller SA, Mentrup T, Shmueli MD, Niemeyer J, Haug-Kroper M, von Blume J, Mayerhofer A, Feederle R, Schröder B. *et al.* Signal Peptide Peptidase-Like 2c (SPPL2c) impairs vesicular transport and cleavage of SNARE proteins. *EMBO Rep* 2019;**20**: e46451.

Sha Y, Liu W, Wei X, Zhu X, Luo X, Liang L, Guo T. Biallelic mutations in Sperm flagellum 2 cause human multiple morphological abnormalities of the sperm flagella (MMAF) phenotype. *Clin Genet* 2019a;**96**:385–393.

Sha Y, Wei X, Ding L, Mei L, Huang X, Lin S, Su Z, Kong L, Zhang Y, Ji Z. DNAH17 is associated with asthenozoospermia and multiple morphological abnormalities of sperm flagella. *Ann Hum Genet* 2019b;**84**:271–279.

Sha Y, Yang X, Mei L, Ji Z, Wang X, Ding L, Li P, Yang S. DNAH1 gene mutations and their potential association with dysplasia of the sperm fibrous sheath and infertility in the Han Chinese population. *Fertil Steril* 2017;**107**:1312–1318. e1312.

Sha YW, Wang X, Su ZY, Mei LB, Ji ZY, Bao H, Li P. Patients with multiple morphological abnormalities of the sperm flagella harbouring CFAP44 or CFAP43 mutations have a good pregnancy outcome following intracytoplasmic sperm injection. *Andrologia* 2019c; **51**:e13151.

Sha YW, Xu X, Mei LB, Li P, Su ZY, He XQ, Li L. A homozygous CEP135 mutation is associated with multiple morphological abnormalities of the sperm flagella (MMAF). *Gene* 2017;**633**: 48–53.

Shamoto N, Narita K, Kubo T, Oda T, Takeda S. CFAP70 is a novel axoneme-binding protein that localizes at the base of the outer dynein arm and regulates ciliary motility. *Cells* 2018;**7**:124.

Shaw-Smith C, Pittman AM, Willatt L, Martin H, Rickman L, Gribble S, Curley R, Cumming S, Dunn C, Kalaitzopoulos D. *et al.* Microdeletion encompassing MAPT at chromosome 17q21.3 is associated with developmental delay and learning disability. *Nat Genet* 2006;**38**:1032–1037.

Shen Y, Zhang F, Li F, Jiang X, Yang Y, Li X, Li W, Wang X, Cheng J, Liu M. *et al.* Loss-of-function mutations in QRICH2 cause male infertility with multiple morphological abnormalities of the sperm flagella. *Nat Commun* 2019;**10**:433.

Stokowy T, Garbulowski M, Fiskerstrand T, Holdhus R, Labun K, Sztromwasser P, Gilissen C, Hoischen A, Houge G, Petersen K. *et al.* RareVariantVis: new tool for visualization of causative variants in rare monogenic disorders using whole genome sequencing data. *Bioinformatics* 2016;**32**:3018–3020.

Sutton KA, Jungnickel MK, Jovine L, Florman HM. Evolution of the voltage sensor domain of the voltage-sensitive phosphoinositide

Human Reproduction, pp. 17–24, 2021
doi:10.1093/humrep/deab099

phosphatase VSP/TPTE suggests a role as a proton channel in eutherian mammals. *Mol Biol Evol* 2012;**29**:2147–2155.

Takeuchi K, Xu Y, Kitano M, Chiyonobu K, Abo M, Ikegami K, Ogawa S, Ikejiri M, Kondo M, Gotoh S. *et al*. Copy number variation in DRC1 is the major cause of primary ciliary dyskinesia in the Japanese population. *Mol Genet Genomic Med* 2020;**8**:e1137.

Tang S, Wang X, Li W, Yang X, Li Z, Liu W, Li C, Zhu Z, Wang L, Wang J. *et al*. Biallelic mutations in CFAP43 and CFAP44 cause male infertility with multiple morphological abnormalities of the sperm flagella. *Am J Hum Genet* 2017;**100**:854–864.

Toure A, Martinez G, Kherraf ZE, Cazin C, Beurois J, Arnoult C, Ray PF, Coutton C. The genetic architecture of morphological abnormalities of the sperm tail. *Hum Genet* 2020;**140**:21–42.

Tu C, Nie H, Meng L, Wang W, Li H, Yuan S, Cheng D, He W, Liu G, Du J. *et al*. Novel mutations in SPEF2 causing different defects between flagella and cilia bridge: the phenotypic link between MMAF and PCD. *Hum Genet* 2020;**139**:257–271.

Tu C, Nie H, Meng L, Yuan S, He W, Luo A, Li H, Li W, Du J, Lu G. *et al*. Identification of DNAH6 mutations in infertile men with multiple morphological abnormalities of the sperm flagella. *Sci Rep* 2019;**9**:15864.

Uhlen M, Oksvold P, Fagerberg L, Lundberg E, Jonasson K, Forsberg M, Zwahlen M, Kampf C, Wester K, Hober S. *et al*. Towards a knowledge-based Human Protein Atlas. *Nat Biotechnol* 2010;**28**:1248–1250.

Visser L, Westerveld GH, Xie F, van Daalen SK, van der Veen F, Lombardi MP, Repping S. A comprehensive gene mutation screen in men with asthenozoospermia. *Fertil Steril* 2011;**95**:1020–1024.e1021–1029.

Wambergue C, Zouari R, Fourati Ben Mustapha S, Martinez G, Devillard F, Hennebicq S, Satre V, Brouillet S, Halouani L, Marrakchi O. *et al*. Patients with multiple morphological abnormalities of the sperm flagella due to DNAH1 mutations have a good prognosis following intracytoplasmic sperm injection. *Hum Reprod* 2016;**31**:1164–1172.

Wang W, Tu C, Nie H, Meng L, Li Y, Yuan S, Zhang Q, Du J, Wang J, Gong F. *et al*. Biallelic mutations in CFAP65 lead to severe asthenoteratospermia due to acrosome hypoplasia and flagellum malformations. *J Med Genet* 2019;**56**:750–757.

Wang X, Jin H, Han F, Cui Y, Chen J, Yang C, Zhu P, Wang W, Jiao G, Wang W. *et al*. Homozygous DNAH1 frameshift mutation causes multiple morphological anomalies of the sperm flagella in Chinese. *Clin Genet* 2017;**91**:313–321.

Whitfield M, Thomas L, Bequignon E, Schmitt A, Stouvenel L, Montantin G, Tissier S, Duquesnoy P, Copin B, Chantot S. *et al*. Mutations in DNAH17, encoding a sperm-specific axonemal outer dynein arm heavy chain, cause isolated male infertility due to asthenozoospermia. *Am J Hum Genet* 2019;**105**:198–212.

Wilson GR, Wang HX, Egan GF, Robinson PJ, Delatycki MB, O'Bryan MK, Lockhart PJ. Deletion of the Parkin co-regulated gene causes defects in ependymal ciliary motility and hydrocephalus in the quakingviable mutant mouse. *Hum Mol Genet* 2010;**19**:1593–1602.

Wirschell M, Olbrich H, Werner C, Tritschler D, Bower R, Sale WS, Loges NT, Pennekamp P, Lindberg S, Stenram U. *et al*. The nexin-dynein regulatory complex subunit DRC1 is essential for motile cilia function in algae and humans. *Nat Genet* 2013;**45**:262–268.

World Health Organization. WHO laboratory manual for the examination and processing of human semen; 2010.

Wu H, Li W, He X, Liu C, Fang Y, Zhu F, Jiang H, Liu W, Song B, Wang X. *et al*. Novel CFAP43 andCFAP44 mutations cause male infertility with multiple morphological abnormalities of the sperm flagella (MMAF). *Reprod Biomed Online* 2019;**38**:769–778.

Wyrwoll MJ, Temel ŞG, Nagirnaja L, Oud MS, Lopes AM, van der Heijden GW, Rotte N, Wistuba J, Wöste M, Ledig S. *et al*. Biallelic mutations in M1AP are a frequent cause of meiotic arrest leading to male infertility. *bioRxiv* 2019;**803346**.

Yzer S, Hollander AI, Lopez I, Pott JW, de Faber JT, Cremers FP, Koenekoop RK, van den Born LI. Ocular and extra-ocular features of patients with Leber congenital amaurosis and mutations in CEP290. *Mol Vis* 2012;**18**:412–425.

Zhang B, Ma H, Khan T, Ma A, Li T, Zhang H, Gao J, Zhou J, Li Y, Yu C. *et al*. A DNAH17 missense variant causes flagella destabilization and asthenozoospermia. *J Exp Med* 2019a;**217**:e20182365.

Zhang X, Shen Y, Wang X, Yuan G, Zhang C, Yang Y. A novel homozygous CFAP65 mutation in humans causes male infertility with multiple morphological abnormalities of the sperm flagella. Clin Genet 2019b;**96**:541–548.
