# Supplementary material for: Exome sequencing reveals variants in known and novel candidate genes for severe sperm motility disorders
Source: Hum Reprod. 2021 Jun 5;36(9):2597–611. doi: 10.1093/humrep/deab099 (PMC8373475; doi:10.1093/humrep/deab099)
Supplement: deab099_Supplementary_TableS2 [file deab099_supplementary_tables2.pdf]

**Human Reproduction, pp. 1–1, 2021**
doi:10.1093/humrep/deab099

human reproduction

SUPPLEMENTARY DATA

**Supplementary Table SII** **Exome filtering statistics.**

| Patient ID | Total variants | Variants present in >5 reads | Variant present in >15% of the reads on that location | Variant frequency In <1% in gnomAD, dbSNP and Nijmegen database | Variants is coding and non-synonymous/ canonical splice site/ synonymous splice site | Homozygous variants (% variation >85) | Compound heterozygous (gene name present >1x) | X-linked (chrX and %variatioon >85) | Y-linked (chrY and % variation > 85) | Qualifying variants in known genes | Homozygous variants in homozygosity region | Variants in gene with enhanced expression in testis | Interacts with DFS-MMAF or infertility gene | Infertility described in mice | Sum of prioritized variants | Likely real variants (Bam file check) | Predicted VUS or higher* |
|---|---|---|---|---|---|---|---|---|---|---|---|---|---|---|---|---|---|
| **ARG1** | 82150 | 63997 | 63349 | 3175 | 685 | 13 | 153 | 2 | 0 | 0 | 0 | 29 | 40 | 4 | 66 | 18 | 4 |
| **ARG2** | 107887 | 89660 | 88712 | 4333 | 641 | 16 | 157 | 5 | 2 | 3 | 0 | 37 | 28 | 7 | 59 | 14 | 3 |
| **ARG3** | 59275 | 50364 | 49752 | 2360 | 571 | 6 | 130 | 5 | 0 | 0 | 0 | 18 | 51 | 2 | 65 | 19 | 4 |
| **ARG4** | 78845 | 61952 | 61276 | 3274 | 636 | 16 | 139 | 4 | 0 | 0 | 4 | 18 | 35 | 3 | 51 | 19 | 3 |
| **ARG5** | 68292 | 51836 | 51289 | 2824 | 661 | 12 | 140 | 7 | 0 | 0 | 2 | 25 | 48 | 5 | 71 | 16 | 1 |
| **ARG6** | 69927 | 56715 | 56128 | 2825 | 611 | 14 | 146 | 10 | 0 | 1 | 1 | 15 | 51 | 10 | 61 | 17 | 2 |
| **ARG7** | 70029 | 56008 | 55369 | 2552 | 612 | 8 | 139 | 4 | 0 | 0 | 0 | 21 | 43 | 7 | 59 | 12 | 6 |
| **ARG8** | 37148 | 36313 | 35555 | 1744 | 669 | 45 | 183 | 7 | 0 | 0 | 30 | 50 | 69 | 21 | 120 | 58 | 21 |
| **ARG9** | 38886 | 38107 | 37366 | 1720 | 645 | 11 | 178 | 7 | 0 | 4 | 0 | 34 | 62 | 16 | 93 | 26 | 6 |
| **AUS1** | 103157 | 72318 | 71407 | 2717 | 620 | 8 | 186 | 9 | 0 | 0 | 0 | 33 | 47 | 5 | 76 | 11 | 6 |
| **AUS2** | 105026 | 74336 | 73440 | 3022 | 670 | 19 | 198 | 6 | 0 | 2 | 2 | 37 | 48 | 7 | 85 | 21 | 7 |
| **AUS3** | 110396 | 80236 | 79341 | 3056 | 585 | 6 | 167 | 8 | 0 | 0 | 0 | 42 | 41 | 5 | 80 | 16 | 11 |
| **AUS4** | 114017 | 84540 | 83539 | 4494 | 852 | 20 | 235 | 12 | 2 | 0 | 1 | 51 | 44 | 6 | 91 | 28 | 9 |
| **AUS5** | 108397 | 78217 | 77273 | 3251 | 635 | 12 | 189 | 2 | 0 | 1 | 1 | 40 | 38 | 7 | 75 | 15 | 3 |
| **AUS6** | 110653 | 80588 | 79707 | 3308 | 660 | 18 | 204 | 4 | 0 | 0 | 1 | 39 | 64 | 12 | 106 | 15 | 1 |
| **AUS7** | 116748 | 86819 | 85905 | 3901 | 776 | 14 | 222 | 11 | 0 | 0 | 1 | 52 | 63 | 3 | 108 | 21 | 4 |
| **AUS8** | 107827 | 76962 | 75940 | 3142 | 652 | 20 | 186 | 7 | 0 | 1 | 2 | 35 | 46 | 5 | 82 | 5 | 1 |
| **AUS9** | 115302 | 87532 | 86427 | 4701 | 947 | 17 | 251 | 13 | 0 | 3 | 2 | 56 | 57 | 3 | 110 | 22 | 1 |
| **AUS10** | 116180 | 87348 | 86312 | 4498 | 857 | 19 | 216 | 5 | 3 | 0 | 0 | 39 | 43 | 5 | 78 | 26 | 2 |
| **AUS11** | 115126 | 85846 | 84819 | 3465 | 674 | 8 | 239 | 6 | 0 | 0 | 0 | 44 | 64 | 13 | 109 | 18 | 3 |
| **AUS12** | 107314 | 78293 | 77445 | 3007 | 649 | 29 | 191 | 10 | 0 | 1 | 17 | 43 | 47 | 9 | 97 | 29 | 10 |
| **Average** | 92503.9 | 70380.3 | 69540.5 | 3208.0 | 681.3 | 15.8 | 183.3 | 6.9 | 0.3 | 0.8 | 3.0 | 36.1 | 49.0 | 7.4 | 83.0 | 20.3 | 5.1 |

*ACMG classification. VUS, variant of unknown significance.
