# Supplementary material for: Exome sequencing reveals variants in known and novel candidate genes for severe sperm motility disorders
Source: Hum Reprod. 2021 Jun 5;36(9):2597–611. doi: 10.1093/humrep/deab099 (PMC8373475; doi:10.1093/humrep/deab099)
Supplement: deab099_Supplementary_TableS3 [file deab099_supplementary_tables3.pdf]

Human Reproduction, pp. 1–5, 2021
doi:10.1093/humrep/deab099

Supplementary Table SIII All prioritized variants.

Patient	Gene	Transcript	gDNA variant	cDNA	Protein	Zygosity	GnomAD variant frequency (population with highest frequency)	Predicted to be pathogenic*	Variant classification (ACMG)**	Gene expression enriched in testis***	Interaction with known infertility gene****	Disease model described (PMID)	Additional information (PMID)	Conclusion
**ARG1**	*DNAH12*	ENST00000351747.2	Chr3(GRCh37):g. 57357584 G > A	c.5393T>C	p.(Phe1798Ser)	Het	0.1% (AFR: 0.4%)	N/A	VUS	Yes	Interacts with known astheno-zoospermia genes *DNAH1* and *DNAH17*	No	-	Novel candidate gene
			Chr3(GRCh37):g. 57407357 A > G	c.7438C>T	p.(Pro2480Ser)	Het	0.0% (AFR: 0.2%)	N/A	VUS					
	POTEC	ENST00000358970.9	Chr18(GRCh37): g.14513699_ 14513702del	c.1492_1495del	p.(Gln498Serfs*3)	Het	0% (AFR: 0.03%)	N/A	VUS	Yes	No	No	-	Unlikely disease causing
	Chr18(GRCh37): g.14538165A>T		c.605T>A	p.(Leu202His)	Het	0% (AFR : 0.01%)	N/A	VUS						
**ARG2**	*CFAP43*	ENST00000357060.7	Chr10(GRCh37): g.105953623C>T	c.1442 + 1G>A	p.?	Het	0.0% (NFE: 0.0%)	N/A	Likely pathogenic	Yes	Interacts with known astheno-zoospermia genes *DNAH1* and *SPEF2*	Yes, mouse (28552195)	Known gene (28552195). c.1040T>C previously seen (29449551)	Disease causing
			Chr10(GRCh37): g.105963506A>G	c.1019T>C	p.(Phe340Ser)	Het (in cis with c.1040T>C)	0.01% (NFE: 0.03%)	–	Unlikely pathogenic					
			Chr10(GRCh37): g.105963485A>G	c.1040T>C	p.(Val347Ala)	Het (in cis with c.1019T>C)	0.01% (NFE: 0.02%)	P	VUS					
**ARG3**	*DNAH6*	ENST00000389394.7	Chr2(GRCh37): g.84775542_ 84775543insC	c.1316 + 1_ 1316 + 2insC	N/A	Het	0.0% (NFE: 0.0%)	N/A	Likely pathogenic	Yes	Interacts with *DNAH1*, *LRRC6*, *DNAH17*, *CCDC40*, *CCDC39*, *PIH1D3*, *WDR66* and *DNAAF2*	Yes, mouse and zebrafish (26918822)	Mutations were previously described in patients with azoospermia (28206990), teratozoospermia characterized by globozoospermia and acephalic spermatozoa (29356036) and heterotaxy (26918822)	Disease causing
			Chr2(GRCh37): g.84926802C>T	c.7762C>T	p.(Arg2588*)	Het	0.0% (AMR: 0.0%)	N/A	Pathogenic					
	PDE6B	ENST00000496514.5	Chr4(GRCh37): g.647752G>A	c.823G>A	p.(Val275Met)	Het	0.0% (EAS: 0.02%)	SMP	VUS	No	Interacts with *CEP290* and *LRRC6*	Yes (mouse), no infertility described	Known gene for Night blindness, congenital stationary, autosomal dominant 2 and Retinitis pigmentosa-40	Unlikely disease causing
	Chr4(GRCh37): g.656373G>A		c.1798G>A	p.(Asp600Asn)	Het	0.0% (SAS: 0.01%)	MP	VUS						
**ARG4**	*PACRG*	ENST00000337019.7	Chr6(GRCh37): g.163483259T>A	c.369T>A	p.(Tyr123*)	Hom	0.00%	N/A	Likely pathogenic	Yes	Interacts with known astheno-zoospermia genes *DNAH17*, *SPEF2*, *DNAAF2*, *CCDC40* and *CCDC39*	Yes, mouse (15148410)	Associated with the development of sperm flagellum (15148410; 25715396)	Novel candidate gene
	LRIT1	ENST00000372105.3	Chr10(GRCh37): g.85997167A>C	c.398T>G	p.(Leu133Arg)	Hom	0.00% (NFE: 0.01%)	SMP	VUS	Yes	No	Yes (mouse), no infertility described	In homozygosity region	Unlikely disease causing
	PAGE3	ENST00000374951.5	ChrX(GRCh37): g.55287068C>T	c.218G>A	p.(Trp73*)	Hem	0.00%	N/A	Pathogenic	Yes	No	No	Gene function unknown	Unlikely disease causing
**ARG5**	*CFAP58*	ENST00000369704.8	Chr10(GRCh37): g.106139973C>T	c.1360C>T	p.(Gln454*)	Hom	0.05% (ASJ: 1.09%)	N/A	Pathogenic	Yes	Interacts with known astheno-zoospermia gene *CCDC40*	No	In homozygosity region Variant is relatively common in Ashkenazi Jewish population	Novel candidate gene
**ARG6**	*CFAP44*	ENST00000393845.9	Chr3(GRCh37): g.113135393del	c.652del	p.(Arg218Aspfs*37)	Hom	0.03% (ASJ: 0.61%)	N/A	Pathogenic	Yes	Interacts with known astheno-zoospermia gene *DNAH1*	Yes, mouse (28552195)	Known gene (28552195)	Disease causing
	MAML2	ENST00000524717.6	Chr11(GRCh37): g.95826041G>A	c.1154C>T	p.(Pro385Leu)	Hom	0.03% (ASJ: 0.55%)	ASMP	VUS	No	Interacts with known infertility gene *MAMLD1*	No	Known for Mucoepidermoid salivary gland carcinoma	Unlikely disease causing
**ARG7**	*DRC1*	ENST00000288710.7	Chr2(GRCh37): g.26637294C>T	c.238C>T	p.(Arg80*)	Het	0.00% (FIN: 0.03%)	N/A	Pathogenic	Yes	Interacts with known astheno-zoospermia genes *CCDC40* *CCDC39*, *LRRC6*, *DNAAF2* and *SPEF2*	Yes, Chlamydomonas reinhardtii (23354437)	Described in primary ciliary dyskinesia (23354437, 31270959)	Novel candidate gene
			Chr2(GRCh37): g.26644264C>T	c.352C>T	p.(Gln118*)	Het	0.04% (0.07% (NFE)	N/A	Pathogenic					
	TRPM1	ENST00000397795.6	Chr15(GRCh37): g.31323230G>A	c.3017C>T	p.(Pro1006Leu)	Het	0.00%	ASMP	VUS	Yes	No	Yes (mouse), no infertility described	Known for night blindness, congenital stationary (complete), 1C, autosomal recessive	Unlikely disease causing
	Chr15(GRCh37): g.31359348G>A		c.470C>T	p.(Ser157Phe)	Het	0.35% (NFE: 0.58%)	ASMP	VUS						
	SDK2	ENST00000392650.8	Chr17(GRCh37): g.71397309G>A	c.2821C>T	p.(Arg941Cys)	Het	0.11% (ASJ: 0.33%)	ASMP	VUS	Yes	No	Yes (mouse), no infertility described	-	Unlikely disease causing
			c.2426G>A	p.(Arg809His)	Het	0.04% (NFE: 0.06%)	SM	VUS						

(continued)

Supplementary Table SIII Continued

Patient	Gene	Transcript	gDNA variant	cDNA	Protein	Zygosity	GnomAD variant frequency (population with highest frequency)	Predicted to be pathogenic*	Variant classification (ACMG)**	Gene expression enriched in testis***	Interaction with known infertility gene****	Disease model described (PMID)	Additional information (PMID)	Conclusion
	Chr17(GRCh37): g.71410841C>T													
**ARG8**	*DNAH6*		**ARG8**	*DNAH6*			ENST00000389394.7	Chr2(GRCh37):	g.84804515C>A					
			c.2059C>A	p.(Pro687Thr)	Hom	0.04% (NFE: 0.07%)	MP	VUS	Yes					
		Interacts with known asthenozoospermia genes *DNAH1*, *LRRC6*, *DNAH17*, *CCDC40*, *CCDC39*, *PIH1D3*, *WDR66* and *DNAAF2*	Yes, mouse and zebrafish (26918822)	Mutations were previously described in patients with azoospermia (28206990), teratozoospermia characterized by globozoospermia and acephalic spermatozoa (29356036) and heterotaxy (26918822)	Novel candidate gene									
	ATP2B4	ENST00000357681.9	Chr1(GRCh37): g.203667467G>C	c.376G>C	p.(Gly126Arg)	Hom	0.01% (SAS: 0.08%)	MP	VUS	No	No	Yes, mouse (15078889)	Mouse displays asthenozoospermia (15078889). In homozygosity region	Novel candidate gene
	CEP350	ENST00000367607.8	Chr1(GRCh37): g.179959750A>G	c.229A>G	p.(Arg77Gly)	Hom	0.34% (ASJ: 0.81%)	ASMP	VUS	No	Interacts with known asthenozoospermia genes *CEP135* and *CEP290*.	No	In homozygosity region	Novel candidate gene
	CEP290	ENST00000552810.6	Chr12(GRCh37): g.88465084T>C	c.5998A>G	p.(Ile2000Val)	Het	0.02% (NFE: 0.04%)	M	VUS	No	Interacts with known asthenozoospermia genes *CEP135* and *PKD1*	Yes, mouse (21623382)	Described in patients with Leber's Congenital Amaurosis and asthenozoospermia (22355252)	Novel candidate gene
	Chr12(GRCh37): g.88519120A>C		c.1092T>G	p.(Ile364Met)	Het	0.08% (SAS: 0.35%)	SMP	VUS						
	ENTPD3	ENST00000301825.7	Chr3(GRCh37): g.40464372C>T	c.863C>T	p.(Pro288Leu)	Hom	0.05% (AMR: 0.13)	ASMP	VUS	No	Interacts with known asthenozoospermia genes *AK7*	Yes (mouse), no infertility described	In homozygosity region	Unlikely disease causing
	CTNND1	ENST00000361391.10	Chr11(GRCh37): g.57563052C>T	c.271C>T	p.(His91Tyr)	Het	0.00%	SMP	VUS	Yes	Interacts with known infertility gene *FGFR1*	No	-	Unlikely disease causing
	Chr11(GRCh37): g.57571209A>G		c.1537A>G	p.(Asn513Asp)	Het	0.05% (AMR: 0.17%)	M	VUS						
	TOR3A	ENST00000367627.8	Chr1(GRCh37): g.179054841A>G	c.452A>G	p.(Tyr151Cys)	Hom	0.00%	ASMP	VUS	No	No	No	In homozygosity region	Unlikely disease causing
	OCLM	ENST00000574641.2	Chr1(GRCh37): g.186370281C>G	c.104C>G	p.(Ser35Cys)	Hom	0.46% (NFE: 0.74%)	N/A	VUS	No	No	No	In homozygosity region	Unlikely disease causing
	SMYD1	ENST00000419482.7	Chr2(GRCh37): g.88409984G>A	c.1426G>A	p.(Glu476Lys)	Hom	0.13% (NFE: 0.22%)	SMP	VUS	No	No	Yes (mouse), no infertility described	In homozygosity region	Unlikely disease causing
	CMC1	ENST00000466830.5	Chr3(GRCh37): g.28361095T>C	c.296T>C	p.(Leu99Pro)	Hom	0.01% (AMR: 0.04%)	MP	VUS	No	No	No	In homozygosity region	Unlikely disease causing
	SEMA6A	ENST00000343348.10	Chr5(GRCh37): g.115840627G>T	c.14C>A	p.(Ala5Asp)	Hom	0.00% (AMR: 0.02%)	ASMP	VUS	No	No	No	In homozygosity region	Unlikely disease causing
	CRCP	ENST00000395326.8	Chr7(GRCh37): g.65595749G>A	c.64G>A	p.(Asp22Asn)	Hom	0.00%	MP	VUS	No	No	No	In homozygosity region	Unlikely disease causing
	ROBO3	ENST00000397801.6	Chr11(GRCh37): g.124740559C>T	c.968C>T	p.(Thr323Met)	Hom	0.23% (SAS: 0.68%)	SMP	VUS	No	Interacts with known infertility gene *SEMA3A*	Yes (mouse), no infertility described	In homozygosity region	Unlikely disease causing
	GCN1	ENST00000300648.7	Chr12(GRCh37): g.120585064A>C	c.4739T>G	p.(Val1580Gly)	Hom	0.00% (NFE: 0.00%)	ASMP	VUS	No	No	No	In homozygosity region	Unlikely disease causing
	CLEC14A	ENST00000342213.2	Chr14(GRCh37): g.38724528G>A	c.700C>T	p.(Arg234Cys)	Hom	0.01% (AFR: 0.05)	ASP	VUS	No	No	No	In homozygosity region	Unlikely disease causing
	SLC12A1	ENST00000396577.7	Chr15(GRCh37): g.48500263G>A	c.347G>A	p.(Arg116His)	Hom	0.34% (OTH: 0.53%)	SMP	VUS	No	Interacts with known infertility gene *CFTR*	No	In homozygosity region	Unlikely disease causing
	CIAO1	ENST00000488633.1	Chr2(GRCh37): g.96934265G>A	c.560G>A	p.(Cys187Tyr)	Hom	0.00% (AFR: 0.01%)	M	VUS	No	Interacts with known infertility gene *WT1* and *NR5A1*	No	–	Unlikely disease causing
	SNW1	ENST00000261531.11	Chr14(GRCh37): g.78184445_78184447del	c.1675_1677del	p.(Arg559del)	Hom	0.00%	N/A	VUS	No	Interacts with known infertility genes AR and *MAMLD1*	No	–	Unlikely disease causing

(continued)

Supplementary Table SIII Continued

Patient	Gene	Transcript	gDNA variant	cDNA	Protein	Zygosity	GnomAD variant frequency (population with highest frequency)	Predicted to be pathogenic*	Variant classification (ACMG)**	Gene expression enriched in testis***	Interaction with known infertility gene****	Disease model described (PMID)	Additional information (PMID)	Conclusion
	COL18A1	ENST00000651438.1	Chr21(GRCh37): g.46925126C>T	c.2947C>T	p.(Arg983Cys)	Hom	0.04% (ASJ: 0.09%)	SP	VUS	No	Interacts with known infertility gene *AR*	Yes (mouse), no infertility described	–	Unlikely disease causing
**ARG9**	*CFAP44*	ENST00000393845.9	Chr3(GRCh37): g.113084927T>C	c.2674A>G	p.(Met892Val)	Het	0.05% (AMR: 0.08%)	–	Likely benign	Yes	Interacts with known astheno-zoospermia gene *DNAH1*	Yes, mouse (28552195)	Known gene (28552195).	Disease causing
			Chr3(GRCh37): g.113098344T>C	c.2107A>G	p.(Arg703Gly)	Het	0.00%	SP	VUS					
			Chr3(GRCh37): g.113098347T>A	c.2104A>T	p.(Ile702Leu)	Het	0.00%	–	Likely benign					
			Chr3(GRCh37): g.113120583A>G	c.1174T>C	p.(Trp392Arg)	Het	0.00%	SMP	VUS					
	UGT2B17	ENST00000317746.2	Chr4(GRCh37): g.69403599A>G	c.1337T>C	p.(Leu446Ser)	Hom	0.25% (AMR: 0.71%)	SP	VUS	No	Interacts with known infertility genes *SRD5A2*, *HSD17B3*, *HSD3B2*, *CYP19A1*, *CYP17A1* and *AR*	No	–	Unlikely disease causing
	F8	ENST00000360256.9	ChrX(GRCh37): g.154158080G>A	c.3985C>T	p.(Arg1329Cys)	Hem	0.01% (NFE: 0.01%)	SP	VUS	No	Interacts with known infertility gene *TEX11*	No	–	Unlikely disease causing
**AUS1**	*TRIP12*	ENST00000283943.9	Chr2(GRCh37): g.230673014C>T	c.2149G>A	p.(Val717Ile)	Het	0.01% (NFE: 0.02%)	MP	VUS	No	Interacts with known infertility gene *TRIM37*	Yes (mouse), no infertility described	-	Unlikely disease causing
			Chr2(GRCh37): g.230701593C>G	c.1115G>C	p.(Arg372Pro)	Het	0.00%	MP	VUS					
	THSD4	ENST00000355327.7	Chr15(GRCh37): g.72057477G>A	c.2708G>A	p.(Ser903Asn)	Het	0.48% (NFE: 0.73%)	M	VUS	No	Interacts with known astheno-zoospermia gene *ARMC2*	No	-	Unlikely disease causing
	Chr15(GRCh37): g.72069595A>G		c.2939A>G	p.(Tyr980Cys)	Het	0.00% (NFE: 0.00%)	SMP	VUS						
	STARD3	ENST00000336308.10	Chr17(GRCh37): g.37815071C>T	c.644C>T	p.(Ala215Val)	Het	0.00%	MP	VUS	No	Interacts with known infertility gene *CYP11A1*	Yes (mouse), no infertility described	-	Unlikely disease causing
	Chr17(GRCh37): g.37816703C>T		c.878C>T	p.(Ala293Val)	Het	0.00% (NFE: 0.00%)	SM	VUS						
**AUS2**	*DNAH1*	ENST00000420323.6	Chr3(GRCh37): g.52397021G>A	c.5105G>A	p.(Arg1702Gln)	Het	0.00% (NFE: 0.00%)	SMP	VUS	No	Interacts with known astheno/ teratozoospermia genes *CFAP43*, *DNAH17*, *DPY19L2*, *SPATA16*, *CFAP44*, *WDR66*, *CCDC40*, *CCDC39*, *DNAAF2*	Yes, mouse (11371505)	Known gene (24360805)	Disease causing
			Chr3(GRCh37): g.52428678G>C	c.10823 + 1G>C	p.?	Het	0.00%	N/A	Likely pathogenic					
	ULK4	ENST00000301831.9	Chr3(GRCh37): g.41723090C>T	c.2887G>A	p.(Val963Met)	Het	0.34% (NFE: 0.48%)	P	VUS	Yes	No	Yes (mouse), no infertility described	Likely on same allele	Unlikely disease causing
	Chr3(GRCh37): g.41831290C>T		c.2056G>A	p.(Val686Ile)	Het	0.35% (NFE: 0.50%)	SP	VUS						
	POLG	ENST00000268124.10	Chr15(GRCh37): g.89868870G>A	c.1760C>T	p.(Pro587Leu)	Het	0.15% (NFE: 0.26%)	MP	VUS	No	Interacts with known infertility gene *CFTR*	Yes, mouse (15164064)	Mouse phenotype does not match MMAF. Variants likely on same allele	Unlikely disease causing
	Chr15(GRCh37): g.89873415G>A		c.752C>T	p.(Thr251Ile)	Het	0.15% (NFE: 0.27%)	M	VUS						
	PNCK	ENST00000447676.6	ChrX(GRCh37): g.152936592T>C	c.917A>G	p.(Gln306Arg)	Hem	0.00%	ASMP	VUS	No	Interacts with known infertility gene *CYP19A1*	No	–	Unlikely disease causing
**AUS3**	*DNAH6*	ENST00000389394.7	Chr2(GRCh37): g.84940276A>G	c.9436A>G	p.(Ser3146Gly)	Het	0.20% (NFE: 0.40%)	MP	VUS	Yes	Interacts with known astheno-zoospermia genes *DNAH1*, *LRRC6*, *DNAH17*, *CCDC40*, *CCDC39*, *PIH1D3*, *WDR66* and *DNAAF2*	Yes, mouse and zebrafish (26918822)	Variants are likely located on the same allele. Mutations were previously described in patients with azoospermia (28206990), teratozoospermia characterized by globozoospermia and acephalic spermatozoa (29356036) and heterotaxy (26918822)	Unlikely disease causing
			Chr2(GRCh37): g.85043186G>A	c.12352G>A	p.(Ala4118Thr)	Het	0.20% (NFE: 0.40%)	MP	VUS					
	LRP1B	ENST00000389484.7	Chr2(GRCh37): g.141116420C>T	c.11227G>A	p.(Gly3743Ser)	Het	0.67% (ASJ: 1.02)	ASMP	VUS	No	Interacts with known infertility gene *DMRT1*	Yes (mouse), no infertility described	-	Unlikely disease causing
			c.9731C>T	p.(Ser3244Leu)	Het	0.00% (AMR: 0.01%)	ASMP	VUS						

(continued)

Supplementary Table SIII Continued

Patient	Gene	Transcript	gDNA variant	cDNA	Protein	Zygosity	GnomAD variant frequency (population with highest frequency)	Predicted to be pathogenic*	Variant classification (ACMG)**	Gene expression enriched in testis***	Interaction with known infertility gene****	Disease model described (PMID)	Additional information (PMID)	Conclusion
	Chr2(GRCh37): g.141215115G>A													
	ESPNL			*ESPNL*			ENST00000343063.8	Chr2(GRCh37):	g.239038829C>G					
			c.1474C>G	p.(Gln492Glu)				Het	0.51% (ASJ: 0.87%)					
		P	VUS	No	Interacts with known MMAF gene *CFAP44* and known infertility gene *PLCZ1*	Yes (mouse), no infertility described	Possibly on same allele. Involved in stereocilia (26926603). Detected in late spermatids	Unlikely disease causing						
	Chr2(GRCh37): g.239040065G>A	c.2710G>A	p.(Val904Met)						Het	0.47% (ASJ: 0.60)	SP	VUS		
	ZSCAN10	ENST00000576985.6	Chr16(GRCh37): g.3139190C>A	c.2245G>T	p.(Ala749Ser)	Het	0.36% (AMR: 0.48%)	–	VUS	No	Interacts with known infertility gene *SOX2*	Yes (mouse), no infertility described	-	Unlikely disease causing
	Chr16(GRCh37): g.3139999G>T		c.1436C>A	p.(Ser479Tyr)	Het	0.36% (AMR: 0.53%)	AS	VUS						
	MCF2	ENST00000519895.5	ChrX(GRCh37): g.138701770C>G	c.963G>C	p.(Leu321Phe)	Hem	0.00% (SAS: 0.03%)	M	VUS	Yes	–	Yes (mouse), no infertility described	–	Unlikely disease causing
	SYP	ENST00000263233.8	ChrX(GRCh37): g.49050648C>T	c.398G>A	p.(Arg133Gln)	Hem	0.00% (AFR: 0.02%)	MP	VUS	No	Interacts with known infertility genes *AR*, *SOX2* and *WT1*	Yes (mouse), no infertility described	–	Unlikely disease causing
	NONO	ENST00000276079.13	ChrX(GRCh37): g.70510568_70510570dup	c.81_83dup	p.(Gln27dup)	Hem	0.00% (AMR: 0.00%)	N/A	VUS	No	Interacts with known infertility gene *AR*	Yes (mouse), no infertility described	–	Unlikely disease causing
**AUS4**	*SPPL2C*	ENST00000329196.6	Chr17(GRCh37): g.43922906C>T	c.634C>T	p.(Arg212Trp)	Hom	0.01% (SAS: 0.06)	S	VUS	Yes	No	Yes, mouse (30733280)	SPPL2c deficiency leads to a partial loss of elongated spermatids and reduced motility of mature spermatozoa, but preserved fertility in mice (30733280). Possibly involved in acrosome formation (30733281)	Novel candidate gene
	HERC2	ENST00000261609.12	Chr15(GRCh37): g.28389261G>A	c.11261C>T	p.(Ala3754Val)	Het	0.00% (SAS: 0.00%)	SMP	VUS	No	Interacts with known asthenozoospermia gene *CEP290* and known infertility gene *TRIM37*	Yes, mouse (7604002, 10441737)	-	Unlikely disease causing
	Chr15(GRCh37): g.28447666G>A		c.7307C>T	p.(Thr2436Met)	Het	0.00% (EAS: 0.03%)	SMP	VUS						
	USH2A	ENST00000307340.8	Chr1(GRCh37): g.216138711A>C	c.7068T>G	p.(Asn2356Lys)	Het	0.08% (EAS: 1.03%)	SMP	VUS	Yes	Interacts with known asthenozoospermia gene *CEP290*	Yes (mouse), no infertility described	Known gene for Retinitis pigmentosa and Usher syndrome	Unlikely disease causing
	Chr1(GRCh37): g.216246607G>A		c.5608C>T	p.(Arg1870Trp)	Het	0.0% (EAS: 0.39%)	SMP	VUS						
	SDK2	ENST00000392650.8	Chr17(GRCh37): g.71334848G>C	c.6397C>G	p.(Arg2133Gly)	Het	0.01% (EAS: 0.15%)	SMP	VUS	Yes	No	Yes (mouse), no infertility described	-	Unlikely disease causing
	Chr17(GRCh37): g.71468262A>G		c.320T>C	p.(Val107Ala)	Het	0.00%	SMP	VUS						
	CLSPN	ENST00000318121.8	Chr1(GRCh37): g.36217053C>T	c.1826G>A	p.(Arg609Lys)	Hom	0.02% (EAS: 0.26%)	SMP	VUS	Yes	No	Yes (mouse), no infertility described	–	Unlikely disease causing
	RPS4Y2	ENST00000629237.1	ChrY(GRCh37): g.22941505G>C	c.643G>C	p.(Gly215Arg)	Hem	0.00%	SP	VUS	Yes	No	No	–	Unlikely disease causing
**AUS5**	*QRICH2*	ENST00000262765.10	Chr17(GRCh37): g.74300558dup	c.145dup	p.(Thr49Asnfs*31)	Hom	0.00% (NFE: 0.00%)	N/A	Pathogenic	Yes	No	Yes, mouse (30683861)	Known gene (30683861, 31292949)	Disease causing
	ANKAR	ENST00000520309.5	Chr2(GRCh37): g.190608005G>A	c.3815G>A	p.(Arg1272His)	Hom	0.11% (NFE: 0.20%)	SMP	VUS	Yes	No	No	In homozygosity region	Unlikely disease causing
	BCORL1	ENST00000540052.5	ChrX(GRCh37): g.129147652C>T	c.904C>T	p.(Pro302Ser)	Hem	0.00% (NFE: 0.01%)	S	VUS	No	Interacts with known infertility gene *WT1*	No	–	Unlikely disease causing
**AUS6**	*MMP19*	ENST00000322569.9	Chr12(GRCh37): g.56231659G>A	c.1028C>T	p.(Ser343Leu)	Hom	0.01% (ASJ: 0.02%)	MP	VUS	No	Interacts with known infertility gene *RSPO1*	Yes (mouse), no infertility described	–	Unlikely disease causing
**AUS7**	*TPTE2*	ENST00000400230.6	Chr13(GRCh37): g.20038622G>A	c.715C>T	p.(Gln239*)	Hom	0.00% (NFE: 0.19%)	N/A	Likely pathogenic	Yes	No	No	Voltage-sensitive phosphatases (22896666)	Novel candidate gene
	OPLAH	ENST00000618853.5	Chr8(GRCh37): g.145109562G>A	c.2588C>T	p.(Thr863Ile)	Het	0.00% (AFR: 0.03%)	P	VUS	Yes	No	Yes (mouse), no infertility described	Associated with 5-oxoprolinase deficiency (23430506)	Unlikely disease causing
			c.157C>T	p.(Arg53Cys)	Het	0.03% (AMR: 0.04%)	SP	VUS						

(continued)

Supplementary Table SIII Continued

Patient	Gene	Transcript	gDNA variant	cDNA	Protein	Zygosity	GnomAD variant frequency (population with highest frequency)	Predicted to be pathogenic*	Variant classification (ACMG)**	Gene expression enriched in testis***	Interaction with known infertility gene****	Disease model described (PMID)	Additional information (PMID)	Conclusion
	Chr8(GRCh37): g.145114779G>A													
	AFF2			*AFF2*			ENST00000370460.6	ChrX(GRCh37):	g.147743457G>A					
			c.209G>A	p.(Arg70Gln)	Hem	0.00% (AFR: 0.02%)	SMP	VUS	No					
		Interacts with known asthenozoospermia gene *AKAP4* and known infertility gene *TEX11*	Yes (mouse), no infertility described	Associated with Mental retardation, X-linked, FRAXE type (8673085)	Unlikely disease causing									
**AUS8**	*CFAP43*	ENST00000357060.7	Chr10(GRCh37): g.105985300T>A	c.335A>T	p.(Asp112Val)	Hom	0.01% (NFE : 0.01%)	SMP	VUS	Yes	Interacts with known asthenozoospermia genes *DNAH1* and *SPEF2*	Yes, mouse (28552195)	Known gene (28552195). In homozygosity region	Disease causing
**AUS9**	*CFAP43*	ENST00000357060.7	Chr10(GRCh37): g.105965740del	c.944del	p.(Gly315Alafs*22)	Hom	0.00%	N/A	Pathogenic	Yes	Interacts with known asthenozoospermia genes *DNAH1* and *SPEF2*	Yes, mouse (28552195)	Known gene (28552195). In homozygosity region	Disease causing
**AUS10**	*SPTA1*	ENST00000643759.1	Chr1(GRCh37): g.158613133C>T	c.4421G>A	p.(Arg1474Gln)	Het	0.00% (EAS: 0.05%)	SMP	VUS	No	Interacts with known infertility gene *TEX15*	Yes, mouse (20056793)	Known gene for elliptocytosis, pyropoikilocytosis and spherocytosis. Unlikely affecting sperm cell morphology	Unlikely disease causing
			Chr1(GRCh37): g.158644127G>A	c.1342C>T	p.(Arg448Trp)	Het	0.02% (AMR: 0.15%)	SP	VUS					
**AUS11**	*MDC1*	ENST00000376406.8	Chr6(GRCh37): g.30679276G>A	c.2134C>T	p.(Gln712*)	Het	0.00%	N/A	Likely pathogenic	Yes	Interacts with known infertility gene AR	Yes, mouse (16427009)	Possibly meiotic defect	Novel candidate gene
			Chr6(GRCh37): g.30681625G>A	c.472C>T	p.(Gln158*)	Het	0.00%	N/A	Likely pathogenic					
	KDM6A	ENST00000377967.8	ChrX(GRCh37): g.44923015A>G	c.1876A>G	p.(Ser626Gly)	Hem	0.00%	SMP	VUS	No	Interacts with known infertility gene *CHD7*, *SOX2*, *GATA4* and *AR*	No	–	Unlikely disease causing
**AUS12**	*QRICH2*	ENST00000262765.10	Chr17(GRCh37): g.74300534C>T	c.169G>A	p.(Glu57Lys)	Hom	0.01% (NFE: 0.02)	P	VUS	Yes	No	Yes, mouse (30683861	Known gene (30683861, 31292949). In homozygosity region	Likely disease causing
	PADI3	ENST00000375460.3	Chr1(GRCh37): g.17603075C>T	c.1369C>T	p.(Pro457Ser)	Hom	0.23% (FIN: 0.58%)	M	VUS	No	No	No	Known gene for Uncombable hair syndrome. In homozygosity region	Unlikely disease causing
	DUSP23	ENST00000368107.1	Chr1(GRCh37): g.159751959G>C	c.284G>C	p.(Cys95Ser)	Hom	0.05% (OTH:"0.08%)	ASMP	VUS	No	No	No	In homozygosity region	Unlikely disease causing
	SLAMF7	ENST00000368043.8	Chr1(GRCh37): g.160721161_160721162delinsAG	c.796_797delinsAG	p.(Asp266Ser)	Hom	0.10% (ASJ: 0.91%)	SP	VUS	No	No	No	In homozygosity region	Unlikely disease causing
	MCF2L2	ENST00000328913.7	Chr3(GRCh37): g.182946129A>C	c.2071T>G	p.(Cys691Gly)	Hom	0.20% (FIN: 0.81%)	SMP	VUS	Yes	No	No	In homozygosity region	Unlikely disease causing
	PTCHD4	ENST00000339488.8	Chr6(GRCh37): g.47846467A>C	c.2113T>G	p.(Leu705Val)	Hom	0.00%	SMP	VUS	No	No	No	In homozygosity region	Unlikely disease causing
	DST	ENST00000244364.10	Chr6(GRCh37): g.56504402G>C	c.1094C>G	p.(Ser365Cys)	Hom	0.00% (NFE: 0.22%)	S	VUS	No	No	No	Known gene for Epidermolysis bullosa simplex, autosomal recessive 2 and Neuropathy, hereditary sensory and autonomic, type VI. In homozygosity region	Unlikely disease causing
	ENDOG	ENST00000372642.5	Chr9(GRCh37): g.131584884_131584887del	c.889_892del	p.(Lys297delinsGluGlyGlyAlaGln)	Hom	0.22% (AMR: 0.67%)	N/A	VUS	No	No	No	In homozygosity region	Unlikely disease causing
	DBH	ENST00000393056.7	Chr9(GRCh37): g.136513028C>T	c.1085C>T	p.(Ala362Val)	Hom	0.22% (FIN: 0.56%)	ASMP	VUS	No	No	No	Known gene for Orthostatic hypotension 1, due to DBH deficiency. In homozygosity region	Unlikely disease causing
	CHRDL1	ENST00000372042.5	ChrX(GRCh37): g.110002913_110002915del	c.275_277del	p.(Pro92del)	Hem	0.01% (NFE: 0.02%)	N/A	VUS	No	Interacts with known infertility gene *APOA1*	No	–	Unlikely disease causing

*A: Align GVGD, S: SIFT, M: MutationTaster, P: PolyPhen-2.
**VUS, Variant of Unknown Significance.
***Based on the Human Protein Atlas version 19.1.
****Based on STRING version 11.0.
