# Supplementary material for: Exome sequencing reveals variants in known and novel candidate genes for severe sperm motility disorders
Source: Hum Reprod. 2021 Jun 5;36(9):2597–611. doi: 10.1093/humrep/deab099 (PMC8373475; doi:10.1093/humrep/deab099)
Supplement: deab099_Supplementary_TableS4 [file deab099_supplementary_tables4.pdf]

**Human Reproduction, pp. 1–1, 2021**
doi:10.1093/humrep/deab099

human reproduction

# SUPPLEMENTARY DATA

**Supplementary Table SIV** Homozygosity regions detected in all patients.

| Sample | Chromosome | Start_positions | End_positions | Length | Variants in region | perc_HMZ |
|---|---|---|---|---|---|---|
| ARG1 | 2 | 160,742,862 | 167,163,043 | 6,420,181 | 151 | 88 |
| ARG1 | 2 | 182,346,954 | 187,455,278 | 5,108,324 | 59 | 87 |
| ARG4 | 6 | 44,253,749 | 56,846,774 | 12,593,025 | 253 | 89 |
| ARG4 | 10 | 85,960,296 | 94,700,460 | 8,740,164 | 152 | 91 |
| ARG4 | 21 | 33,730,128 | 43,824,123 | 10,093,995 | 332 | 93 |
| ARG6 | 2 | 218,683,139 | 223,918,080 | 5,234,941 | 194 | 92 |
| ARG6 | 6 | 44,268,371 | 51,619,808 | 7,351,437 | 85 | 88 |
| ARG8 | 1 | 177,929,554 | 221,055,463 | 43,125,909 | 380 | 92 |
| ARG8 | 2 | 99,804,642 | 118,575,215 | 18,770,573 | 123 | 87 |
| ARG8 | 3 | 14,106,310 | 49,726,028 | 35,619,718 | 257 | 95 |
| ARG8 | 3 | 49,829,326 | 75,786,440 | 25,957,114 | 187 | 92 |
| ARG8 | 3 | 119,118,104 | 183,212,026 | 64,093,922 | 355 | 92 |
| ARG8 | 5 | 89,990,324 | 134,344,497 | 44,354,173 | 199 | 89 |
| ARG8 | 6 | 150,004,779 | 166,571,935 | 16,567,156 | 149 | 91 |
| ARG8 | 7 | 42,970,733 | 56,087,319 | 13,116,586 | 96 | 90 |
| ARG8 | 11 | 121,403,229 | 129,794,950 | 8,391,721 | 133 | 89 |
| ARG8 | 12 | 118,682,751 | 131,296,880 | 12,614,129 | 138 | 88 |
| ARG8 | 13 | 95,097,956 | 115,047,464 | 19,949,508 | 158 | 92 |
| ARG8 | 14 | 21,623,648 | 50,120,858 | 28,497,210 | 245 | 91 |
| ARG8 | 15 | 31,320,703 | 70,345,626 | 39,024,923 | 422 | 90 |
| ARG8 | 22 | 36,124,860 | 45,204,519 | 9,079,659 | 161 | 89 |
| ARG8 | 22 | 45,931,262 | 51,137,249 | 5,205,987 | 107 | 98 |
| AUS4 | 11 | 49,054,028 | 56,143,544 | 7,089,516 | 106 | 87 |
| AUS5 | 2 | 172,339,964 | 207,621,759 | 35,281,795 | 845 | 97 |
| AUS5 | 10 | 62,540,165 | 72,432,521 | 9,892,356 | 201 | 93 |
| AUS5 | 13 | 52,536,114 | 60,707,316 | 8,171,202 | 68 | 86 |
| AUS5 | 16 | 46,696,284 | 53,913,911 | 7,217,627 | 93 | 88 |
| AUS7 | 9 | 13,112,116 | 18,680,350 | 5,568,234 | 135 | 85 |
| AUS8 | 2 | 151,858,101 | 159,651,973 | 7,793,872 | 179 | 88 |
| AUS8 | 10 | 91,373,847 | 118,397,971 | 27,024,124 | 904 | 95 |
| AUS12 | 1 | 7,805,208 | 12,853,805 | 5,048,597 | 250 | 93 |
| AUS12 | 1 | 17,313,454 | 22,832,743 | 5,519,289 | 412 | 96 |
| AUS12 | 1 | 113,933,570 | 120,612,040 | 6,678,470 | 186 | 93 |
| AUS12 | 1 | 152,192,687 | 161,495,885 | 9,303,198 | 677 | 96 |
| AUS12 | 3 | 140,185,367 | 154,859,581 | 14,674,214 | 217 | 92 |
| AUS12 | 3 | 179,439,567 | 186,390,332 | 6,950,765 | 262 | 92 |
| AUS12 | 6 | 41,657,328 | 57,246,785 | 15,589,457 | 542 | 95 |
| AUS12 | 6 | 62,887,031 | 80,631,539 | 17,744,508 | 248 | 93 |
| AUS12 | 9 | 119,977,077 | 137,004,907 | 17,027,830 | 1,031 | 95 |
| AUS12 | 10 | 63,520,698 | 73,846,826 | 10,326,128 | 371 | 91 |
| AUS12 | 13 | 36,805,625 | 51,969,383 | 15,163,758 | 385 | 91 |
| AUS12 | 14 | 96,771,959 | 105,170,003 | 8,398,044 | 376 | 93 |
| AUS12 | 17 | 67,309,039 | 75,199,580 | 7,890,541 | 480 | 92 |
| AUS12 | 18 | 21,703,649 | 50,683,691 | 28,980,042 | 416 | 93 |
