# Supplementary material for: Exome sequencing reveals variants in known and novel candidate genes for severe sperm motility disorders
Source: Hum Reprod. 2021 Jun 5;36(9):2597–611. doi: 10.1093/humrep/deab099 (PMC8373475; doi:10.1093/humrep/deab099)
Supplement: deab099_Supplementary_TableS5 [file deab099_supplementary_tables5.pdf]

Human Reproduction, pp. 1–2, 2021
doi:10.1093/humrep/deab099

human reproduction

SUPPLEMENTARY DATA

Supplementary Table SV **Frequency of heterozygous and homozygous missense and loss of function variants in 22 known sperm tail assembly and 6 candidate genes in a cohort of 5784 proven fathers.**

Gene name	Loss of function		Rare missense	
	Heterozygous	Homozygous	Heterozygous	Homozygous
AK7	3	0	126	0
AKAP3	2482	206*	126	0
AKAP4	0	0	0	0
ARMC2	4	0	122	0
CFAP100	77	0	272	1
CFAP126	2	0	38	0
CFAP157	9	0	77	0
CFAP161	0	0	25	0
CFAP20	0	0	5	0
CFAP206	12	0	249	0
CFAP221	10	0	125	0
CFAP36	5	0	46	0
CFAP43	12	0	489	0
CFAP44	12	0	384	3
CFAP45	3	0	174	0
CFAP46	126	1*	1031	6
CFAP47	0	0	0	0
CFAP52	4	0	82	0
CFAP53	6	0	93	2
CFAP54	35	0	468	3
CFAP57	8	0	295	0
CFAP58	6	0	122	2
CFAP61	51	0	234	1
CFAP65	18	0	426	4
CFAP69	7	1*	146	1
CFAP70	2	0	111	0
CFAP73	16	0	63	0
CFAP74	52	0	550	1
CFAP77	0	0	165	0
CFAP99	75	0	230	4
DNAH1	25	0	1241	4
DNAH10	27	1*	911	5
DNAH10OS	2	0	31	0
DNAH11	2879	1426*	1123	5
DNAH12	49	0	498	4
DNAH14	907	28*	993	11
DNAH17	26	0	1136	6
DNAH2	72	0	960	5
DNAH3	46	0	975	13
DNAH5	42	0	808	3
DNAH6	50	0	668	1
DNAH7	177	1*	973	12

(continued)

Supplementary Table SV Continued

Gene name	Loss of function		Rare missense	
	Heterozygous	Homozygous	Heterozygous	Homozygous
DNAH8	121	1*	848	1
DNAH9	274	14*	752	4
DRC1	16	0	208	0
DZIP1	1	0	147	1
FSIP2	33	0	1349	9
MAATS1	21	0	120	0
MDC1	29	0	412	5
PACRG	15	0	74	0
QRICH2	17	0	343	0
SPEF2	42	0	623	8
SPPL2C	0	0	283	0
TPTE2	31	0	68	0
TTC21A	19	0	181	0
WDR66	31	0	315	2

*Loss of function variants identified in (potentially) flagellar genes:

– AKAP3:
  ○ Variant: Chr12(GRCh37):g.4735966dup; NM_001278309.1(AKAP3):c.2102dup; p.(Asp702Argfs4)
  ○ Classification: benign
  ○ Wrongfully called as a LoF variant and is present in 12% of the gnomAD population.
– CFAP46:
  ○ Variant: Chr10(GRCh37):g.134623929G>A; NM_001200049.2(CFAP46):c.7648C>T; p.(Arg2550*)
  ○ Classification: uncertain significance
  ○ Nonsense variants in the penultimate exon. It is predicted not to result in nonsense-mediated decay and is therefore a doubtful loss-of-function variant
– CFAP69:
  ○ Variant: Chr7(GRCh37):g.89906394del; NM_001039706.2(CFAP69):c.992del; p.(Gly331Alafs6)
  ○ Classification: pathogenic
  ○ It is unknown whether this proven father underwent ICSI, but it does show that homozygous LoF variants in CFAP69 likely do not cause sterility
– DNAH10:
  ○ Variant: Chr12(GRCh37):g.124383395_124383449dup; NM_207437.3(DNAH10):c.9286+34_9286+88dup; p.?
  ○ Classification: pathogenic
  ○ It is unknown whether this proven father underwent ICSI, but it does show that homozygous LoF variants in DNAH10 likely do not cause sterility
– DNAH11:
  ○ Variant: Chr7(GRCh37):g.21582963G>T; NM_001277115.1(DNAH11):c.100G>T; p.(Glu34)
  ○ Classification: benign
  ○ Variant is located in first exon of DNAH11, the presence of an alternative start site is possible. Variant present in 44% of gnomAD.
– DNAH14:
  ○ Various variants including: Chr1(GRCh37):g.225273315del; NM_001367479.1(DNAH14):c.3397del; p.(Met1133Cysfs16) and Chr1(GRCh37):g.225380567_225380570del; NM_001367479.1(DNAH14):c.5840_5843del; p.(Lys1947Thrfs*6)
  ○ Classification of variants: likely benign/benign
  ○ Various homozygous LoF variants present in proven fathers. This gene is very unlikely to be involved in male infertility
– DNAH7:
  ○ Variant: Chr2(GRCh37):g.196851911del; NM_018897.2(DNAH7):c.1633del; p.(Arg545Valfs2)
  ○ Classification: likely pathogenic
  ○ It is unknown whether this proven father underwent ICSI, but it does show that homozygous LoF variants in DNAH7 likely do not cause sterility
– DNAH8:
  ○ Variant: Chr6(GRCh37):g.38738337T>C; NM_001206927.1(DNAH8):c.1764+2T>C; p.?
  ○ Classification: benign
  ○ This is a canonical slice donor site mutation. The variant is predicted to disrupt splicing, but it is unclear if this is indeed the consequence. Variant is present in 7.2% of the African population in gnomAD
– DNAH9:
  ○ Variant: Chr17(GRCh37):g.11501859_11501883dup; NM_001372.3(DNAH9):c.44_68dup; p.(Arg24Glyfs36)
  ○ Classification: likely benign
  ○ Variant is located in first exon of DNAH9, the presence of an alternative start site is possible. Variant present in 1.3% of gnomAD.
